# Structural and Biochemical Analysis Reveals Catalytic Mechanism of Fucoidan Lyase from *Flavobacterium* sp. SA-0082

**DOI:** 10.3390/md20080533

**Published:** 2022-08-20

**Authors:** Juanjuan Wang, Zebin Liu, Xiaowei Pan, Ning Wang, Legong Li, Yuguang Du, Jianjun Li, Mei Li

**Affiliations:** 1 Division of Life Sciences and Medicine, University of Science and Technology of China, Hefei 230027, China; 2 National Laboratory of Biomacromolecules, CAS Center for Excellence in Biomacromolecules, Institute of Biophysics, Chinese Academy of Sciences, Beijing 100101, China; 3 State Key Laboratory of Biochemical Engineering, Institute of Process Engineering, Chinese Academy of Sciences, Beijing 100190, China; 4 College of Life Science, Capital Normal University, Beijing 100101, China

**Keywords:** fucoidan lyase, polysaccharides, crystal structure, catalytic mechanism

## Abstract

Fucoidans represent a type of polyanionic fucose-containing sulfated polysaccharides (FCSPs) that are cleaved by fucoidan-degrading enzymes, producing low-molecular-weight fucoidans with multiple biological activities suitable for pharmacological use. Most of the reported fucoidan-degrading enzymes are glycoside hydrolases, which have been well studied for their structures and catalytic mechanisms. Little is known, however, about the rarer fucoidan lyases, primarily due to the lack of structural information. FdlA from *Flavobacterium* sp. SA-0082 is an endo-type fucoidan-degrading enzyme that cleaves the sulfated fuco-glucuronomannan (SFGM) through a lytic mechanism. Here, we report nine crystal structures of the catalytic N-terminal domain of FdlA (FdlA-NTD), in both its wild type (WT) and mutant forms, at resolutions ranging from 1.30 to 2.25 Å. We show that the FdlA-NTD adopts a right-handed parallel β-helix fold, and possesses a substrate binding site composed of a long groove and a unique alkaline pocket. Our structural, biochemical, and enzymological analyses strongly suggest that FdlA-NTD utilizes catalytic residues different from other β-helix polysaccharide lyases, potentially representing a novel polysaccharide lyase family.

## 1. Introduction

Fucoidans are a class of sulfated, fucose-rich polysaccharides produced by brown algae and certain marine invertebrates [1,2]. The backbone of fucoidans is generally linked via an α-1,3- and/or α-1,4-glycosidic bond and is highly variable in length and monosaccharide composition. In addition to fucose (Fuc), fucoidans also contain galactose (Gal), mannose (Man), glucuronic acid (GlcUA), and other types of monosaccharide [3]. Moreover, the L-fucose residues in fucoidans are usually sulfated at different hydroxyl group positions, including C-2, C-3, and C-4 [4]. The diverse composition in monosaccharides, the variation in sulfate ester pattern and content, and the different branching sites for sugar chains result in considerable structural variation among fucoidans produced by different brown algae [5,6]. Furthermore, the structural complexity of fucoidans is influenced by other factors, including the geographical locations of macroalgal species, the specific time of harvest of brown algae, as well as the methods used for isolation and purification of fucoidans [7].

Fucoidans represent a suitable candidate drug possessing antiviral activity, as they were recently reported to effectively inhibit SARS-CoV-2 [8]. It was shown that fucoidans tightly bind to the S-protein of SARS-CoV-2, thus acting as a decoy that interferes with the binding of the S-protein to the heparin sulfate co-receptor present at the surface of host cells, potentially inhibiting viral infection [8]. In addition to their antiviral activity, fucoidans show antithrombotic, anticoagulant, anti-inflammatory, antitumor, and immunomodulatory effects [9,10]. One report indicated that the sulfate patterns (the sulfate content and the position of the sulfate groups) present on fucoidans are important for their bioactivity [11]. However, native fucoidans are usually characterized by high molecular weight, high viscosity, and irregular structures, considerably hindering their application as therapeutic agents. In contrast, low-molecular-weight fucoidans (LMWFs) are easily absorbed and possess higher bioavailability, rendering these polysaccharides a more promising target for pharmaceutical use [9,12]. Therefore, depolymerization of HMWFs into LMWFs using specific enzymes is a suitable approach, as it preserves the integrity of the specific structure of fucoidans and generates relatively homogeneous degradation products.

Fucoidan-degrading enzymes are promising tools for producing bioactive fucoidan oligosaccharides for a range of biomedical applications [13,14]. Fucoidan-degrading enzymes differ in their mode of action and are usually classified into exo- or endo-enzymes [12]. Exo-fucoidan-degrading enzymes are capable of cleaving fucoidans from the terminus of the sugar chain, usually producing monosaccharides. Endo-fucoidan-degrading enzymes break the glycosidic bond from the middle of the sugar chain to produce oligosaccharides exhibiting different degrees of polymerization [15]. Currently, all fucoidan-degrading enzymes that have been identified act as endo-hydrolases, and are classified into glycoside hydrolases (GHs) family 107 (GH107, endo-α-1,4-L-fucanase (EC 3.2.1.212)), and 168 (GH168, endo-α-(1,3)-L-fucanase (EC 3.2.1.211)) in the Carbohydrate Active enZymes database (CAZy database, http://www.cazy.org, accessed on 1 July 2022) [16,17]. The marine bacterium *Flavobacterium* sp. SA-0082 was earlier reported to produce a novel type of extracellular endo-fucoidan lyase that cleaves the sulfated fucoglucuronomannan from *Kjellmaniella crassifolia* (Kj-fucoidan) [18,19]. Two genes that encode for putative fucoidan lyase have been identified in the genome of this marine bacterium (*Flavobacterium* sp. SA-0082), and their gene products were termed FdlA and FdlB [19]. These two enzymes are 56% identical at the amino acid sequence level. The enzymatic activity of FdlA is higher than that of FdlB when acting on Kj-fucoidan [20].

**Scheme 1:**
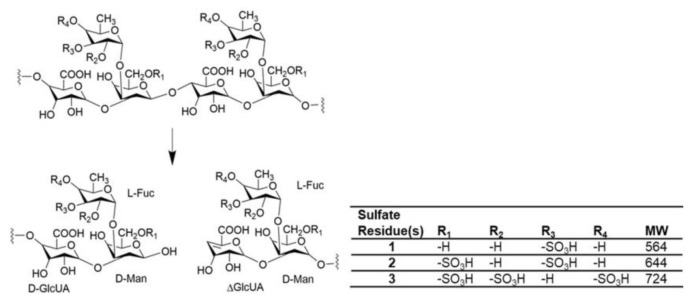
Enzymatic reaction catalyzed by FdlA to digest Kj-fucoidan and yield three types of trisaccharides.

Earlier studies carried out biochemical characterization and enzymatic analysis for native FdlA, demonstrating that the optimal conditions for catalytic activity of FdlA are a temperature of 40 °C, slightly alkane pH of pH 7.5, as well as the presence of NaCl at 0.4 M concentration [12,21]. Previous studies found that polysaccharides lacking sulfated fucose are not cleaved by native FdlA, indicating that the sulfated fucoses of fucoidans are important components for their recognition and cleavage by the lyase [19]. The final products of Kj-fucoidan cleaved by FdlA were identified as three types of trisaccharides, characterized by an identical backbone structure termed Δ^4,5^Glc*p*UAβ1-2L-Fuc*p*α1-3D-Man*p*. Nevertheless, these trisaccharide molecules possess different numbers of sulfate groups and/or different sulfation positions, namely monosulfated (Molecular weight (Mw) 564 Da, Fuc*p*(3-*O*-sulfate)), disulfated (Mw 644 Da, Fuc*p*(3-*O*-sulfate)α1-3D-Man*p*(6-*O*-sulfate)), and trisulfated (Mw 724 Da, Fuc*p*(2,4-*O*-disulfate)α1-3D-Man*p*(6-*O*-sulfate)) trisaccharides (Scheme 1), with the monosulfated form being the major degradation product [21]. Based on the structures of the final products, it can be deduced that FdlA acts on the α-1,4-linkage between D-mannose and D-glucuronic acid in Kj-fucoidan, which possesses a branched sulfated fucose linked on the C-3 hydroxyl group of D-mannose [5,21]. However, despite the extensive body of biochemical and enzymological analysis, the precise catalytic mechanisms responsible for FdlA activity remain elusive.

To date, FdlA has not been classified into any enzyme family in the CAZy database. However, as it acts as a uronic acid-containing polysaccharide lyase, it presumably belongs to the family of polysaccharide lyases (PL). At present, 42 PL families have been identified in the CAZy database, and are grouped into six classes based on their overall folding, namely into the right-handed parallel β-helix class (termed β-helix henceforth), the (α/α)_n_ barrel class, the β-jelly roll class, the β-propeller class, the β-sandwich and β-sheet class, as well as the triple-stranded β-helix class [22]. Members from the same PL family show high sequence similarity and possess essentially conserved catalytic residues. However, FdlA exhibits low amino acid sequence homology with all these PL members, rendering it impossible to classify FdlA into the existing PL families without information about its structure and key catalytic residues [5,23].

Despite possessing different folds, all PLs degrade uronic acid-containing polysaccharides via a β-elimination mechanism, utilizing Brønsted base and acid to cleave the scissile glycosidic bond [24]. This reaction process yields a new non-reducing end with an unsaturated bond in the sugar products. The catalytic mechanisms of PLs are generally divided into two groups based on the neutralizers used for the C-5 carboxyl group: (i) His/Tyr β-elimination and (ii) metal ion (usually Ca^2+^)-assisted β-elimination. In the former type, amino acid residues are used as a neutralizer, and a histidine and a tyrosine usually act as Brønsted base and acid, respectively. In the latter, a metal ion serves as a neutralizer, and an arginine and a lysine commonly act as Brønsted base and acid, respectively [25]. One previous report found that metal ions are unable to stimulate FdlA activity [19], suggesting that FdlA adopts the His/Tyr β-elimination mechanism for catalysis. However, the key residues for catalysis and their locations in FdlA remain unclear.

While previously obtained structural and enzymatic data provide important information for understanding the structure and function of fucoidan hydrolases and polysaccharide lyases, the overall folding and the catalytic mechanism of fucoidan lyases remain to be elucidated. Here, we report crystal structures and enzymological characterization of wild type (WT) and eight single mutants of the catalytic N-terminal domain (NTD) of FdlA, revealing its unique substrate binding pocket and identifying the key catalytic residues. Our results provide detailed structural information on fucoidan lyase and therefore should facilitate a deeper understanding of the catalytic mechanisms used by uronic polysaccharide lyases.

## 2. Results

### 2.1. Purification and Biochemical Characterization of FdlA-NTD

As shown in Figure S1, the full-length FdlA from *Flavobacterium* sp. SA-0082 contains 697 amino acid residues including a secretory signal peptide (Met1–Thr24). Based on the conserved domain database (CDD), the C-terminal region (residue 472–697) of FdlA contains an F5/8 type C domain (known as the discoidin domain) and a por secretion tail (known as a secretion system C-terminal sorting domain) [26,27], thus it may not be related to FdlA lyase activity, while its NTD is likely to constitute the catalytic domain of FdlA. This assumption was supported by sequence alignment of FdlA and FdlB, another fucoidan lyase from *Flavobacterium* sp. SA-0082. This result showed that both enzymes contain an N-terminal domain with a high sequence identity of approximately 75% (Figure S1).

As the full-length FdlA tends to aggregate when expressed in *E. coli*, we constructed a truncated version of FdlA solely consisting of the NTD (FdlA-NTD, residue 25–471), and successfully expressed and purified the truncated recombinant enzyme (Figure S2A,B). Size exclusion chromatography indicated that this FdlA-NTD form exists in solution as monomers. Circular dichroism (CD) analysis showed that FdlA-NTD is stable at temperatures below 35 °C for at least 120 min, while its secondary structure starts to change at 40 °C following a short incubation of 10 min (Figure S2C). This observation suggested that FdlA-NTD is characterized by low heat resistance. Furthermore, our FdlA-NTD form exhibited superior pH stability under neutral and alkaline pH conditions, preserving its secondary structure at pH 6.0–11.0, even after extended incubation of 17 h (Figure S2D).

### 2.2. Enzymatic Properties of FdlA-NTD

**Figure 1:**
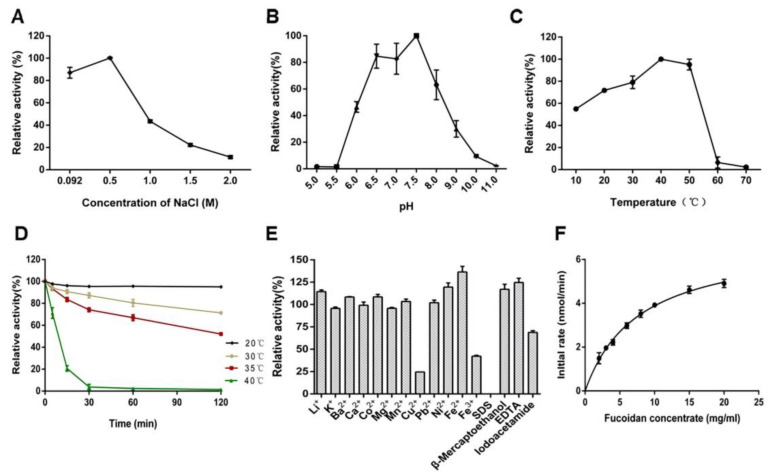
Biochemical and enzymatic characterization of FdlA-NTD using Kj-fucoidan as the substrate. (**A**) Effects of NaCl on FdlA-NTD activity. (**B**) Effects of pH on FdlA-NTD activity. (**C**) Effects of temperature on FdlA-NTD activity. (**D**) The kinetics of the thermal inactivation of FdlA-NTD at 20 °C (black diamond), 30 °C (yellow circle), 35 °C (red square) and 40 °C (green triangle). (**E**) Effects of metal ions and chemical reagents on FdlA-NTD activity. (**F**) The catalytic kinetics of FdlA-NTD. Error bars represent standard deviation (n = 3 independent experiments).

We next evaluated the activity of FdlA-NTD under various conditions by measuring the 232 nm absorption of the products of Kj-fucoidan degraded by FdlA-NTD (Figure 1), according to a previously described method [19]. FdlA-NTD shows relatively high salt tolerance, displaying the highest activity in the presence of 0.5 M NaCl (Figure 1A). The optimal pH and temperature for catalytic activity of FdlA-NTD were pH 7.5 and 40 °C after 1 min-incubation (Figure 1B,C). However, we found that FdlA-NTD activity was greatly decreased after incubation at 40 °C for 5 min and its enzymatic activity was almost completely lost following 30 min-incubation (Figure 1D). Our enzymatic data were consistent with the CD results obtained for FdlA-NTD (Figure S2C). On the basis of these findings, we next performed all enzyme reactions under the optimal conditions but at room temperature (25 °C) instead of 40 °C.

Enzymes of the PL family are commonly found to be associated with metal ions, especially Ca^2+^. These ions participate in the catalytic step through a metal-dependent β-elimination mechanism. One earlier study demonstrated that native FdlA does not require metal ions for catalysis [19]; therefore, we tested the effect of metal ions and a variety of chemical reagents on the enzymatic activity of FdlA-NTD. As shown in Figure 1E, most metal ions, including Ca^2+^ and Ba^2+^, failed to affect the catalytic activity of FdlA-NTD, while Cu^2+^ and Fe^3+^ greatly inhibited the catalytic activity of FdlA-NTD. A small number of chemical reagents, including EDTA, slightly stimulated FdlA-NTD activity. Together, these results strongly suggested that the activity of FdlA-NTD, similar to that of the native full-length protein, is independent of metal ions [19]. On the basis of these findings, we concluded that the truncated FdlA-NTD possesses identical enzymatic properties as its full-length native protein, and it may adopt the His/Tyr elimination mechanism for catalysis. 

We next determined the kinetic parameters of the catalytic reaction of recombinant FdlA-NTD with Kj-fucoidan concentrations ranging from 0.2 to 2% (*w*/*v*) for 1 min, under the optimized conditions (Figure 1F), using the Lineweaver-Burk equation [28]. The kinetic values obtained were only the apparent parameters, as saturation of Kj-fucoidan towards FdlA-NTD was not reached, even at an almost saturated concentration of Kj-fucoidan. The calculated apparent Km and kcat values of FdlA-NTD towards Kj-fucoidan were 7.7 ± 0.5 mg/mL and 59.0 ± 4.3 s^−1^, respectively, resulting in the kinetic efficiency (kcat/Km) of 7.66 ± 0.011 mL/mg/s.

### 2.3. Analysis of Degradation Products of the FdlA-NTD

**Figure 2:**
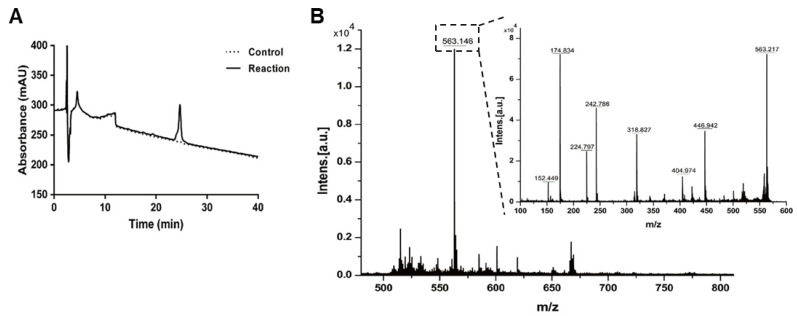
Identification of products of the Kj-fucoidan digested by FdlA-NTD. (**A**) The HPLC purification of Kj-fucoidan products degraded by FdlA-NTD. (**B**) Mass spectrum (negative ESI-MS) of the products of Kj-fucoidan cleaved by FdlA-NTD. The secondary MS result is shown in the inset.

To identify the degradation products of FdlA-NTD, we purified its degraded oligosaccharide products through high-performance liquid chromatography (HPLC) before characterizing these products by mass spectrometry (MS) (Figure 2). Analysis of the primary MS of peak at *m/z* 563.146 (Figure 2B). The molecular weight was consistent with a monosulfated trisaccharide, the major trisaccharide product previously reported for native FdlA [21]. Further analysis of the secondary MS of the peak at *m/z* 563.146 identified the exact structure of the degradation product as △^4,5^Glc*p*UAβ1-2(L-Fuc*p*(3-*O*-sulfate)α1-3)D-Man*p*, matching well with the previously the digested products showed that the major degradation product corresponded to the reported monosulfated trisaccharide product. 

### 2.4. Overall Structure of FdlA-NTD

We solved the crystal structure of FdlA-NTD WT at 1.3 Å resolution using the single-wavelength anomalous diffraction (SAD) method (Table S1). In the FdlA-NTD structure, two molecules are present in an asymmetric unit and adopt nearly identical conformations, with a root-mean-squares deviation (RMSD) of 0.11 Å for all Cα atoms (Figure S3). 

**Figure 3:**
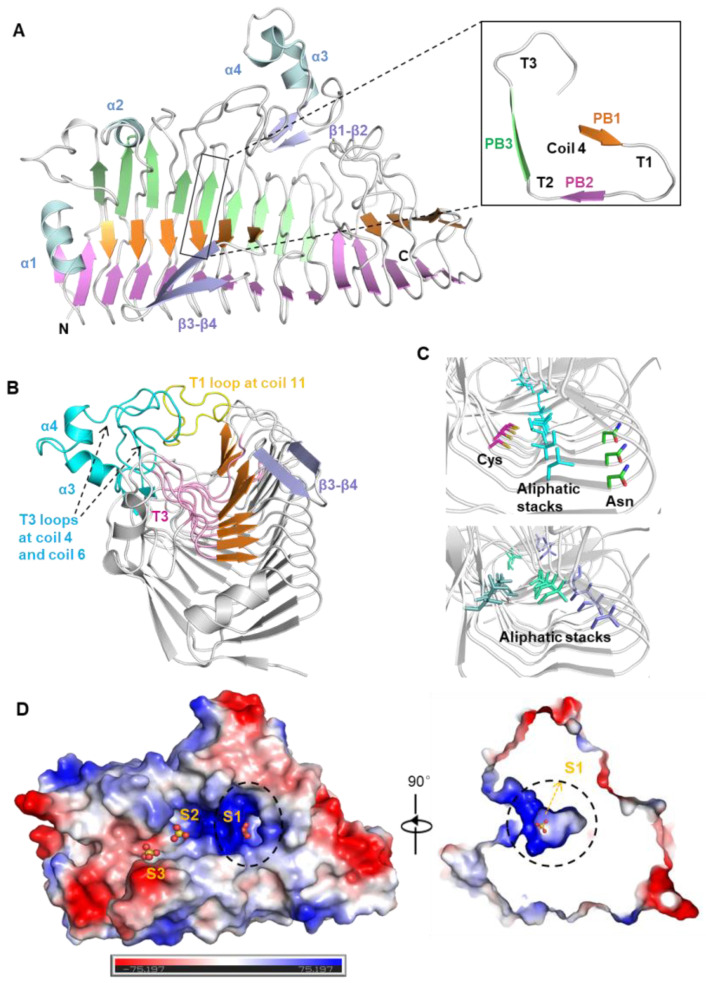
Overall structure of FdlA-NTD. (**A**) Cartoon representation of FdlA-NTD. The PB1 (orange), PB2 (violet), PB3 (green), and accessory elements, including four α helices (α1–α4, light cyan) and two pairs of antiparallel strands (β1–β2 and β3–β4, slate) are indicated. Coil 4 as a representative coil of β-helix structure was shown in the inset. (**B**) The groove formed by PB1 (orange) and T3 (light pink), and enclosed by α3–α4, β1–β2 and T3 loops from coils 4 and 6 (cyan) at one side, β3–β4 (slate) at the opposite side, and the T1 loop from coil 11 (yellow) at the C-terminal end. (**C**) The unusual cysteine ladder as well as four aliphatic stacks and a short asparagine ladder located inside the β-helix structure of FdlA-NTD. The residues constitute various ladders and stacks are shown as sticks, and in different colors for carbon atoms. (**D**) Electrostatic surface representation of FdlA-NTD (blue and red represent the positive and negative charge, respectively). Three sulfate groups in the ‘groove-pocket’ region are shown in ball-and-stick mode. S1 is located in the alkaline pocket at the C-terminal end of the groove (marked by dotted circle).

FdlA-NTD forms a right-handed parallel β-helix (Figure 3A), one type of the six PL classes, shaping like a fish skeleton with a dimension of 67 Å × 22 Å × 47 Å. Here, we adopted the nomenclature of Yoder and Yurnak in describing the β-helix structure [29]. The β-helix fold comprises three parallel β-sheets, PB1, PB2, and PB3, together with three turns (T) linking two β-strands, T1 (between PB1 and PB2), T2 (between PB2 and PB3), and T3 (between PB3 and PB1). In most β-helix proteins, PB1 and PB2 form an antiparallel β sandwich, while PB3 is positioned nearly perpendicular to PB2. The regular β helix unit can be regarded as one coil with a specific order of PB1-T1-PB2-T2-PB3-T3 (Figure 3A).

The FdlA-NTD molecule contains 13 coils, with coils 1–6 being complete, while the other coils lacking either PB1 or PB3. The T3 loops at coils 1–9 together with the adjacent PB1 sheets form a 30-Å long concave groove at the surface of the β-helix (Figure 3B). PB2s and PB3s are located at the bottom face of the β-helix, beneath PB1s and T3s, respectively, supporting the shape of the groove from below.

In addition, FdlA-NTD possesses several accessory elements, including four α-helices (α1–α4) and two pairs of antiparallel strands (β1–β2 and β3–β4) (Figure 3A). The α1 is an amphipathic helix lying at the N-terminal end of the β-helix, with the hydrophobic region facing towards the β-helical interior. Two helices (α3 and α4) as well as one pair of antiparallel strands (β1–β2) are located at the T3 loop of coil 5. Together with the long flexible T3 loops at coil 4 and coil 6, these fragments form a side wall at one side of the groove, while the other pair of antiparallel strands (β3–β4) inserts into the T1 loop of coil 6, and shapes the opposite side-wall of the groove. At the C-terminal end, the groove is sealed by the T1 loop, which is located in coil 11 and protrudes upwards (Figure 3B). This structural arrangement suggests that these accessory elements play a pivotal role in encircling the elongated groove, and this semi-open groove presumably constitutes the substrate binding site for FdlA-NTD.

### 2.5. Structural Elements for FdlA-NTD Stabilization

A β-helix fold usually contains a number of characteristic residues whose side-chains are stacked or aligned either within the interior, or sometimes at the exterior space, of the β-helix [29,30,31]. These residues greatly contribute to the stabilization of β-helix proteins and are generally divided into three types: asparagine ladders, aliphatic stacks (comprising mainly residues Val, Ile, and Leu), and aromatic stacks [32]. When analyzing the FdlA-NTD structure, we identified four aliphatic stacks and one short asparagine ladder (Figure 3C). The longest aliphatic stack on PB2 is located opposite the other three shorter aliphatic stacks lying on PB1 and PB3, and contains 12 hydrophobic residues located on coils 2–13, almost spanning across the entire molecule. In addition to these common stacks and ladders, we also found an unusual cysteine ladder composed of five cysteine residues (Cys168, Cys192, Cys267, Cys290, Cys309) that are located at the T2-PB3 juncture of coils 4–8 (Figure 3C), beneath the potential substrate binding groove. Together, these stacks and ladders stabilize the β-helix folding of FdlA-NTD.

### 2.6. FdlA Uses a Unique Positively-Charged ‘Groove-Pocket’ for Substrate Binding

Enzymes belonging to five PL families (PL1, PL3, PL6, PL9, and PL31) adopt right-handed parallel β-helix folding, similar to FdlA-NTD. We compared our FdlA-NTD structure with lyase structures selected from each of the five families (PL1-pectate lyase: 2ewe [30]; PL3-pectate lyase: 4z04 and 4ew9 [33]; PL6-alginate lyase: 6itg and 6a40 [34]; PL9- pectate lyase: 5olq and 5olr [35]; PL31-alginate lyase: 6kfn [36]), and found that FdlA-NTD failed to superimpose well with these PLs (r.m.s.d of 2.5–3.6 Å for all Cα atoms). Besides this, FdlA-NTD exhibits low sequence homology (lower than 15%) and possesses non-conserved catalytic residues with all other structurally characterized PL proteins (Figure S4). 

One common feature of all these β-helix PLs is that they possess a surficial groove that was previously shown to be the substrate binding groove of these PLs [30,33,34,35,36]. However, the T1 and T3 loops of these enzymes exhibit a wide range of lengths and adopt different conformations, resulting in the different sizes and shapes of these grooves (Figure S4). These structural variations may facilitate specific substrate recognition, in agreement with the fact that these enzymes differ in their substrate selectivity.

In contrast to other PL enzymes, which form a planar groove, FdlA-NTD extends its C-terminal part of the surficial groove into a deep pocket, measuring 11 Å × 13 Å × 8 Å (Figure 3D). The region comprising the deep pocket and the C-terminal part of the groove (between coil 5 and coil 9) is characterized by a strong positive charge (Figure 3D), which is unfavorable for metal binding due to the electrostatic repulsion. This feature is in agreement with the results of our enzymatic assays, showing that FdlA-NTD catalysis is independent of metal ions. In agreement with this result, the β-helix PLs (from PL1, PL3, PL9, and PL31) adopting metal-dependent elimination mechanism possesses an acidic active region (Figure S4), which presumably stabilizes the binding of metal ions. 

Moreover, we observed several bulbs of non-protein densities in the ‘groove-pocket’ region, and modeled these densities as three sulfate groups (S1–S3) (Figure 3D and Figure S5A), based on the density shape and the fact that high concentration of sulfate ammonium was added in the crystallization reagent. One sulfate S1 is located in the alkaline pocket (Figure 3D), strongly indicating that the positively charged pocket facilitates the attraction and interaction with the negative charged sulfate groups of fucoidans, explaining the previous observation that FdlA can only cleave fucoidans containing sulfated fucoses. We docked a monosulfated trisaccharide into the FdlA-NTD structure and found that the alkaline pocket can well accommodate the trisaccharide (Figure S5B), thus the pocket may act as an anchor to grasp the bent sugar chain tightly into the ‘groove-pocket’ region. Compared with a planar groove, the ‘groove-pocket’ mode is more suitable for the branched polysaccharide chain and thus may play an essential role in substrate recognition and stabilization. Such a structural design can be very efficient in the degradation of high molecular weight polysaccharides with branched chains.

### 2.7. Enzyme-Substrate Docking Model Reveal the Catalytic Site

**Figure 4:**
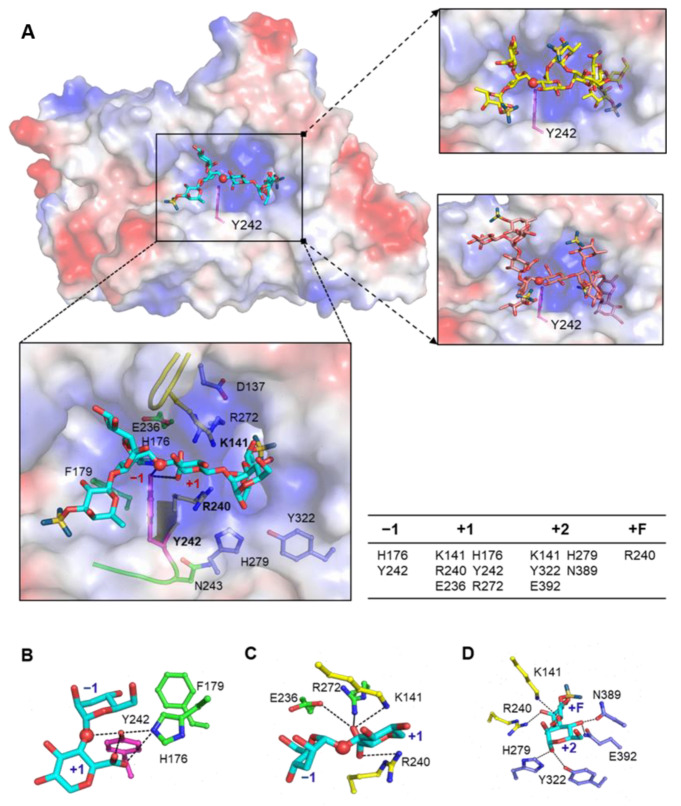
Docking models of FdlA-NTD with three types of oligosaccharides. (**A**) Docking models of FdlA-NTD with hexasaccharide (FdlA-NTD-HS, cyan), nonasaccharide (FdlA-NTD-NS, yellow), and dodecasaccharide (FdlA-NTD-DS, deep salmon). Oligosaccharides are shown as sticks, the glycosidic O atom at the putative cleavage site is highlighted as a red ball. Residue Tyr242 is shown as magenta sticks and indicated. The bottom box shows the residues of FdlA-NTD potentially interacting with HS in the FdlA-NTD-HS docking model. Residues are shown as sticks in different colors according to their locations. Residues close to Tyr242 (His176, Phe179, Glu236, and Asn243) are shown as green sticks for carbon atoms, residues shaping the alkaline pocket (Asp137, Arg272, His279, and Tyr322) are shown as blue sticks for carbon atoms, residues Lys141 and Arg240 near the +1 subsite are shown as yellow sticks for carbon atoms. Residues involved in the interaction with different subsites of HS are listed. (**B**) The potential interactions between Tyr242, His176, and HS. Glycosidic O atom (red ball) and carboxyl group at +1 subsite are hydrogen-bonded with Tyr242. (**C**) Interactions between residues of FdlA-NTD and the C-5 carboxyl group at +1 subsite of HS. (**D**) Interactions between residues of FdlA-NTD and the +2 and +F subsites of HS.

As we failed to obtain the structures of FdlA-NTD in complex with substrate or product or their analogues, we then docked [37] representative substrate oligosaccharides, namely hexasaccharide (HS), nonasaccharide (NS), and dodecasaccharide (DS) ((△^4,5^Glc*p*UAβ1-2(L-Fuc*p*(3-*O*-sulfate)α1-3)D-Man*p*)-α1-4Glc*p*UAβ1-2(L-Fuc*p*(3-*O*-sulfate)α1-3)D-Man*p*)_1-3_, into the FdlA-NTD structure, and established three docking models, namely FdlA-NTD-HS, FdlA-NTD-NS, and FdlA-NTD-DS (Figure 4). When we analyzed the FdlA-NTD-HS model, we observed that one trisaccharide unit is accommodated in the alkaline pocket, while another trisaccharide unit is bound within the groove. In FdlA-NTD-NS and FdlA-NTD-DS, models of FdlA-NTD binding to longer oligosaccharides, we found that the first trisaccharide unit is deeply inserted inside the pocket, and the second trisaccharide is positioned at the opening of the pocket. The remaining one or two trisaccharide units are located within the groove (Figure 4A). These results confirmed our hypothesis that the alkaline pocket is responsible for binding a trisaccharide unit of the sugar substrate. Interestingly, the sulfate groups of different substrates are not located at the same position within the alkaline pocket, which is probably because the entire pocket is positively charged, thus enabling the sulfate group of trisaccharides to bind at multiple sites. This observation also explains the finding of a previous study, showing that the native FdlA is able to degrade fucoidans of different sulfated levels and produce three types of trisaccharides [21].

Previous results suggested that the site within the oligosaccharides that is attacked by FdlA is the glycosidic O (C-4 oxygen) atom between two neighboring trisaccharide units [21]. Our docking models showed that these oligosaccharides contain two trisaccharides located at similar positions, independent of their length. The glycosidic O atom between the two units can be well superimposed among three docking models (Figure 4A), suggesting this C-4 oxygen atom represents the cleaved site. According to the nomenclature of sugar-binding subsites proposed by Davies et al. [38], the monosaccharides are numbered as the subsite +1, +2, +3, +n, starting from the cleaved site to the reducing end of the polysaccharides, and −1, −2, −3, −n to the non-reducing end of the polysaccharides. The proceeding of β-elimination catalysis requires neutralization of the C-5 carboxyl group at +1 subsite. The carboxyl groups in the three docking models are also located at similar places. In addition, both glycosidic O atoms between −1 and +1 subsites and the carboxyl group at +1 subsite are hydrogen bonded with Tyr242 (Figure 4A), implying the essential role of Tyr242 for FdlA-NTD catalysis. Tyr242 in the substrate binding groove is located at the edge of the alkaline pocket (Figure 4A). This arrangement, together with our observation that the pocket is able to accommodate a trisaccharide unit (Figure S5B), strongly suggests that Tyr242 directly participates in cleaving the substrate to produce trisaccharides, and the alkaline pocket assists in orientating the polysaccharide substrate into a position close to Tyr242. This suggestion is in line with findings from our enzymatic analysis that the cleaved products of FdlA-NTD are exclusively trisaccharides (Figure 2).

### 2.8. Residues Essential for Enzymatic Activity of FdlA-NTD

**Figure 5:**
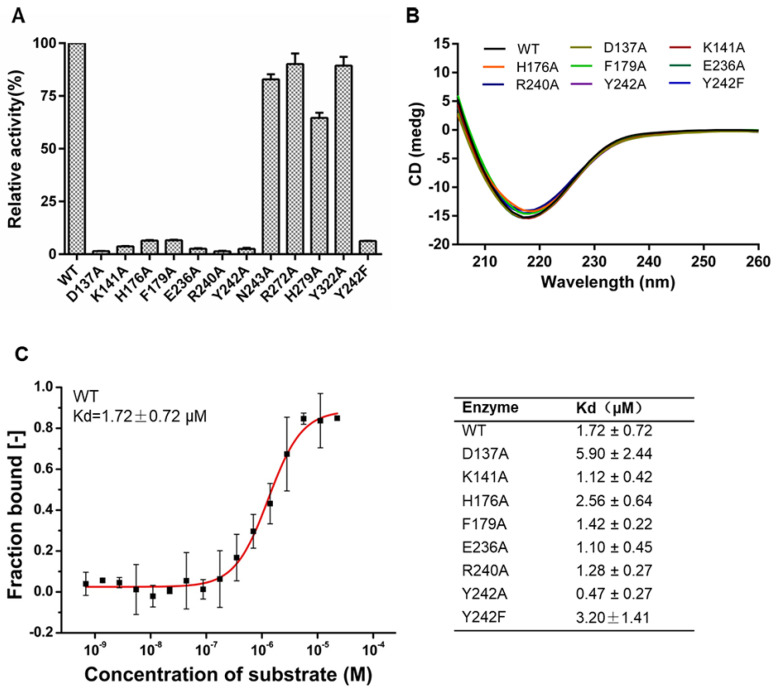
Characterization of FdlA-NTD mutants. (**A**) Enzymatic activities of the WT and mutant forms of FdlA-NTD towards Kj-fucoidan. (**B**) CD spectra of the WT and mutant forms of FdlA-NTD. (**C**) Binding affinity of FdlA-NTD (wild type and inactive mutants) with the substrate (Kj-fucoidan). The representative result of WT with Kj-fucoidan is shown.

Based on our crystal structure and docking models, we selected 11 residues potentially essential for FdlA-NTD activity (Figure 4), and generated 12 single mutants, by replacing each of the 11 residues with alanine as well as mutating Tyr242 to phenylalanine (Figure S2B). We first measured the enzymatic activity of 12 mutants (Figure 5A) and found that eight of them (D137A, K141A, H176A, F179A, E236A, R240A, Y242A, and Y242F) almost completely abolished their activities, H279A mutant retained approximately 65% of the activity compared with WT, while mutations of Asn243, Arg272, and Tyr322 to Ala only negligibly affect their activities. These results are consistent with our docking models, which showed that Asn243, Arg272, and Tyr322 are located slightly distant from the substrate compared with other residues (Figure 4A). Circular dichroism (CD) spectra showed that all inactive mutants possess similar secondary structures to the wild-type protein (Figure 5B), indicating that the loss of activity of these mutants is due to the residue mutations, but not protein conformational changes.

To assess whether these mutations affect the interaction between FdlA-NTD and the polysaccharide substrate, we next measured the binding affinity of FdlA-NTD (wild type and inactive mutants) with the substrate (Kj-fuoicdan) using the MicroScale Thermophoresis (MST) method (Figure 5C and Figure S6). We found that the inactive FdlA-NTD mutants exhibited similar K_d_ values as the wild-type form. These binding assay data clearly demonstrated that while our inactive mutants lost their catalytic activity, they maintained their substrate binding ability. This experimental observation is in agreement with the fact that the substrate of FdlA-NTD is a type of high molecular weight macromolecule, hence it may form multiple interactions with the protein. Thus, a single mutation of the enzyme does not significantly affect its binding affinity with the substrate.

### 2.9. Crystal Structures of FdlA-NTD Mutants

**Figure 6:**
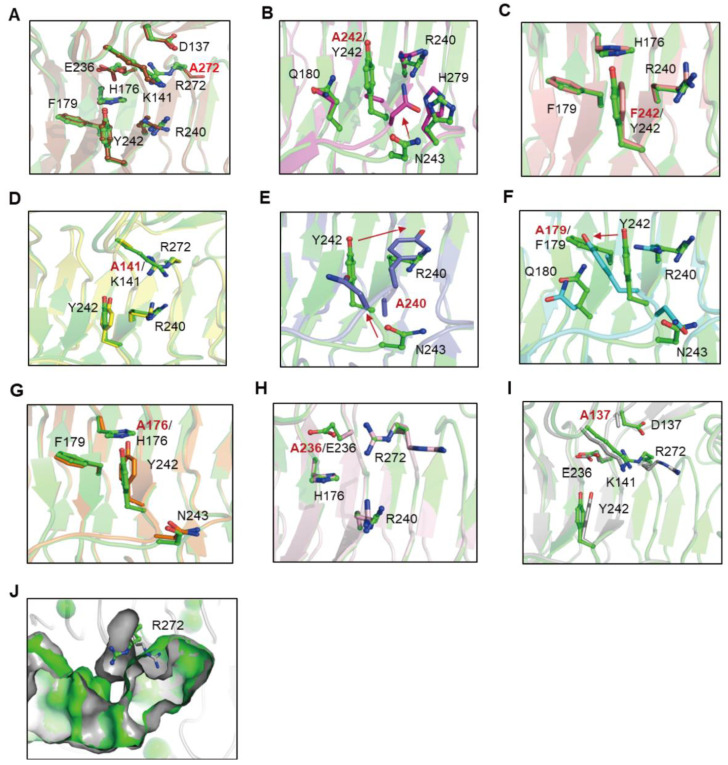
Comparison between the active sites of the inactive mutants and WT of FdlA-NTD. (**A**–**I**) Comparison of mutants R272A (**A**), Y242A (**B**), Y242F (**C**), K141A (**D**), R240A (**E**), F179A (**F**), H176A (**G**), D236A (**H**), D137A (**I**) with WT. Key residues are shown as sticks. The carbon atoms are shown in green for WT in (**A**–**I**), brown for R272A in (**A**), magenta for Y242A in (**B**), pink for Y242F in (**C**), yellow for K141A in (**D**), slate for R240A in (**E**), cyan for F179A in (**F**), orange for H176A in (**G**), light pink for D236A in (**H**) and gray for D137A in (**I**). Residues are labeled with the mutated residues labeled in red. (**J**) The side chain of Arg272 (gray stick) in D137A mutant (grey surface) partially occupies the deep substrate pocket present in WT (green surface).

To analyze the potential function of these residues, we further solved 11 crystal structures of FdlA-NTD mutants (eight inactive and three active mutants) except for H279A, which yielded no crystals (Figure S7, Table S1). The overall structures and the putative active region of the three active mutants (N243A, R272A, and Y322A) are nearly identical to that of the WT (Figure 6A, R272A as a representative), well explaining how full activities of these mutants were maintained. Furthermore, we found that the inactive mutants failed to exhibit significant structural changes compared with the wild type, which is consistent with our CD results (Figure 5B). However, we identified changes around the potential active site in several inactive mutants (Figure 6), implying the potential roles of these residues in catalysis.

Mutation of Y242A resulted in a large structural change at the potential active site (Figure 6B and Figure S7A), with a 3 Å shift of Asn243, which narrows the substrate groove in the mutant structure. However, the Y242F mutant, which also lost its catalytic activity, possesses a potential active site nearly identical to the wild-type form (Figure 6C and Figure S7B). This result highlighted the importance of the hydroxyl group of the Tyr242 side chain. In our docking models of FdlA-NTD with fuco-oligasaccharide, the hydroxyl group of Tyr242 side chain is located within a hydrogen bond distance with the glycosidic O atom between −1 and +1 subsites (Figure 4A), thus allowing Tyr242 to donate a proton to glycosidic O atom and function as a catalytic acid. This suggestion was supported by a previous report showing that a conserved tyrosine residue in β-helix PL31 family members serves as the Brønsted acid [36].

Brønsted base is responsible for proton extraction from C-5 atom in β-elimination reaction [39]. Two positively charged residues Lys141 and Arg240 have their side chains pointing closely to the C-5 atom at the +1 subsite (Figure 4C), hence are potential candidates for a catalytic base. Our structures showed that the K141A mutant possesses an active site almost identical to the WT (Figure 6D and Figure S7C), similar to the Y242F mutant. In contrast, the R240A mutant exhibits visible structural changes around the active site. Specifically, the Try242 residue was rotated approx. 90 degrees, and shifted toward Ala240 (Figure 6E and Figure S7D). As it constitutes the catalytic acid, the hydroxyl group of the Tyr242 side chain is essential for the enzymatic activity, and its improper positioning may severely affect the catalysis of the enzyme. These structures suggested that the loss of activity of the R240A mutant may be due to the shift of the Tyr242 side chain. In contrast, Lys141 possibly functions as a catalytic base by capturing a proton from the C-5 atom at +1 subsite. 

Similar to R240A, the mutant form F179A also exhibited a structural rearrangement around the active site, with a 3.8 Å shift of the Tyr242 side chain towards Ala179 (Figure 6F and Figure S7E). Residue His176 is located close to Tyr242, and the H176A mutation shows a slight influence on the Tyr242 side chain conformation (Figure 6G and Figure S7F). Therefore, mutation of Phe179 and His176 possibly results in the dysfunction of Tyr242 and hence loss of the catalytic activity of FdlA-NTD. In addition, His176 forms a hydrogen bond (2.8 Å) with the hydroxyl group of Tyr242 side chain in WT (Figure 4B), implying another possibility that it might play a role in providing a proton to Tyr242 to facilitate the catalytic reaction.

In the WT structure, both Asp137 and Glu236 form hydrogen bonds with Arg272, while Arg272 participates in the shaping of the alkaline pocket (Figure 6H–J). In both D137A and E236A mutant structures, the side chain of Arg272 switches to the opposite direction compared with that in WT due to the loss of the acidic residue (Arg272 in E236A mutant also exhibits an alternative conformation similar to that in WT), and partially occupies the deep substrate pocket (Figure 6J and Figure S7G,H). These results suggest that the steric hindrance of Arg272 in the two mutants may interfere with the substrate fully entering the pocket and hence the correct positioning of the glycosidic O atom, thus resulting in the complete loss of activities of D137A and E236A mutants. This hypothesis is supported by the fact that the R272A mutation does not significantly affect the enzymatic activity, as this mutant form possesses an identical active site (Figure 6A) hence a similar pocket compared to that of the WT form.

## 3. Discussion

In this study, we purified recombinant FdlA-NTD and identified its cleaved product of Kj-fucoidan as the monosulfated trisaccharide, which is the same as the major product of native FdlA [21]. However, native FdlA produced two additional types of trisaccharides using Kj-fucoidan as substrate, all of which contained an identical backbone with the monosulfated trisaccharide product but carried a different number of sulfate groups. One possible explanation for this observation is that the substrate (Kj-fucoidan) that FdlA and FdlA-NTD cleave exhibits slightly different structures, resulting in different product profiles for FdlA-NTD and FdlA. The Kj-fucoidan used in the previous study was isolated from brown algae harvested in the sea of Hokkaido, Japan. In contrast, the Kj-fucoidan used in our work was obtained from brown algae growing near Dalian, China. Despite being harvested from the same species, the different geographical locations of brown algae may result in the production of fucoidan characterized by different structures/sulfated levels [4], which may account for the small discrepancy of the enzymatic products between the present work and the earlier report.

We performed an enzymatic assay on FdlA-NTD and identified its optimal catalytic conditions. In addition, we showed that the sulfhydryl reagent (iodoacetamide) reduces the activity of FdlA-NTD (Figure 1E). An earlier report showed similar results for native FdlA, implying that FdlA constitutes a sulfhydryl enzyme [19]. However, our crystal structure of FdlA-NTD suggested that it is not a sulfhydryl enzyme, as the five cysteine residues are actually located beneath the hypothetical substrate binding groove (Figure 3C). Thus, the cysteine residues may not directly participate in the catalysis, but rather help to stabilize the groove from the bottom by forming the cysteine ladder. Therefore, the inhibition of FdlA/FdlA-NTD by sulfhydryl reagents may be due to the conformational disturbance upon the modification of the cysteine ladder.

FdlA-NTD possibly belongs to the β-helix PL family; however, its unique structural features, as well as the non-conserved nature of its catalytic residues compared with other known PLs with β-helix fold suggested that FdlA constitutes a novel β-helix PL family hitherto not identified. In addition, FdlA may use unique catalytic residues functioning as Brønsted base and acid for the β-elimination catalysis. While it remains considerably challenging to unambiguously identify residues responsible for proton abstraction and transfer without structures of FdlA-NTD in complex with substrates or products, we were able to infer from our crystal structures and docking models that Tyr242 and Lys141 serve as the potential Brønsted acid and base, respectively. 

Our biochemical data demonstrated that FdlA-NTD lyase favors the β-elimination catalytic mechanism independent of metal ions, and thus is likely to use positively charged amino acid residues to neutralize the negative charge of the substrate. Usually, more than one residue functions as a neutralizer in PLs [40], thus the potential neutralizer residues are difficult to identify, since mutation of a single neutralizer residue may not greatly affect its activity. Based on our structural and docking models, several positively charged residues such as Arg240, His176, and Lys141, located near the +1 subsite, potentially serve as neutralizers in FdlA-NTD, weakening the negative charge of the C-5 carboxyl group at +1 subsite, and making the C-5 proton susceptible to the attack by a Brønsted base.

**Figure 7:**
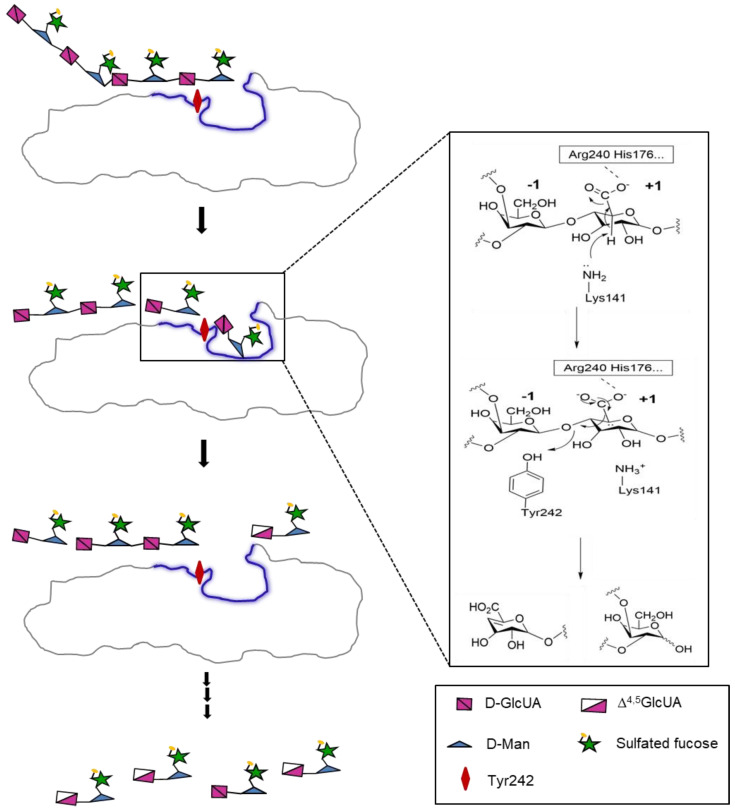
Proposed catalytic model of FdlA-NTD towards Kj-fucoidan. Schematic of the FdlA-NTD and kj-fucoidan were shown. The positively-charged region of the ‘groove-pocket’ of FdlA-NTD was colored blue. During catalysis, the positively charged residues (such as Arg240 and His176) stabilize the negatively charged carboxylate group at +1 subsite, thus facilitating the depriving of the proton at the C-5 position (+1 subsite) by a catalytic base (possibly Lys141). Tyr242 may serve as a catalytic acid to donate a proton, then the glycosidic bond between −1 and +1 subsites are broken and generate a 4,5-unsaturated sugar at the new non-reducing end of the product.

On the basis of our structure and biochemical analysis of wild-type and mutant proteins combined with docking models, we propose the following model describing the catalytic process of FdlA (Figure 7). First, the substrate binds in the groove of the enzyme and the binding is stabilized through multiple interactions. One trisaccharide unit is inserted into the deep alkaline pocket, which is critical for substrate recognition and the proper positioning of the cleaved glycosidic bond. Next, the positively charged residues including Arg240 and His176, which are located near the +1 subsite, neutralize the C-5 carboxyl group of D-GlcUA at +1 subsite, thus reducing the pKa of the C-5 proton, making it more susceptible to the attack by a base. Subsequently, abstraction of the proton occurs on C-5, a process that is presumably accomplished by Lys141, leading to the formation of an enolate intermediate. The hydroxyl group of the Tyr242 side chain is directed to the glycosidic oxygen of the scissile bond where it may function as a Brønsted acid to break the glycosidic bond between −1 and +1 subsites, generating a 4,5-unsaturated sugar at the new non-reducing end of the product. Lastly, a new catalytic cycle is repeated after the cleaved products leave the putative active site.

## 4. Materials and Methods

### 4.1. Materials

Unless specified, all chemicals are of analytical grade and were purchased from Sigma (St. Louis, DE, USA) or Aladdin (Shanghai, China). *Escherichia coli* Trans10 and BL21 (DE3) were purchased from TransGen Biotech (Beijing, China). Crystallization screen commercial kits were purchased from Hampton Research (Aliso Viejo, CA, USA). Ni^2+^-NTA column, Superdex^TM^ 200 10/300 GL, and DEAE Sepharose Fast Flow were purchased from GE Life Sciences and GE Healthcare ((Chicago, IL, USA). Fucoidan from *Kjellmaniella crassifolia* used in this experiment was given by Professor Qiukuan Wang of Dalian Ocean University (Dalian, China).

### 4.2. Cloning, Expression, and Purification

The codon-optimized sequence of full-length FdlA encoding a fucoidan lyase (fucoglucuronomannan lyase) from *Flavobacterium* sp. SA-0082 (GenBank^TM^ accession number AAO00510.1) was synthesized by Shanghai Sangon Biotech Co. Ltd. (Shanghai, China). Sequence analysis showed that the NTD of FdlA is likely the catalytic domain. Therefore, the cDNA encoding FdlA-NTD (residue 25–471) without signal peptide was cloned into pET28a between the *Nde* I and *Xhol* I restriction sites, with an N-terminal 6 × his tag. All point mutations of FdlA-NTD were generated through the QuickChange site-directed mutagenesis method (Stratagene Ltd., La Jolla, CA, USA) by overlap-PCR [41].

The above plasmids were transformed into *Escherichia coli* BL21 (DE3) and the clones were cultured in Lucia-Bertani (LB) medium containing 25 μg/mL Kannamycin at 37 °C until the OD_600nm_ reached 0.6–0.8. Isopropyl β-D-1-thiogalactopyranoside (IPTG) was then added at a final concentration of 1 mM and the cells were cultured at 18 °C for an additional 18 h. The cells were harvested and resuspended in lysis buffer (20 mM Hepes pH 7.5, 400 mM NaCl, 5% glycerol, 10 mM imidazole). After sonication, the cell lysate was centrifuged at 18,000 rpm for 30 min at 4 °C and the supernatant was loaded onto the Ni^2+^-NTA affinity column. The target protein was eluted by imidazole of 300 mM concentration and further purified through size-exclusion chromatography (Superdex^TM^ 200 10/300 GL colume) in a buffer containing 20 mM Hepes pH 7.5, 100 mM NaCl, 5% glycerol. The purity of the enzyme was analyzed via SDS-PAGE in 12% polyacrylamide gels.

To obtain the selenomethionine (SeMet)-labeled FdlA-NTD (FdlA-NTD^SeMet^), the protein was expressed in *E. coli* B834 (DE3). The cells grown overnight in LB medium were harvested when the OD_600nm_ reached 0.6–0.8 and then transferred into M9 medium supplemented with various amino acids (60 mg/liter L-SeMet, 100 mg/liter L-lysine, L-threonine, L-phenylalanine; 50 mg/liter L-leucine, L-isoleucine, L-valine). The cells were incubated at 37 °C for 40 min, then cooled to 16 °C for 30 min and IPTG was added. Finally, the culture was incubated at 16 °C for 18 h. The purification steps of FdlA-NTD^SeMet^ were the same as those of the native protein described above.

### 4.3. Crystallization, Data Collection, and Structure Determination

Crystals were grown at 18 °C through the sitting-drop vapor diffusion method by mixing 0.7 μL of the protein solution (14 mg/mL) with an equal volume of various reservoir solutions. FdlA-NTD crystals were formed in a reservoir solution containing 0.1 M CAPS, pH 10.5, 0.2 M Li_2_SO_4_, 2 M (NH_4_)_2_SO_4_, 1% (*v*/*v*) Pluronic F-68 at 18 °C. Crystals of FdlA-NTD^SeMet^ were obtained under the same conditions without Pluronic F-68. Crystals of mutants D137A and E236A were grown in a reservoir solution containing 0.1 M Tris, pH 8.5, 0.2 M Li_2_SO_4_, 30% (*w*/*v*) polyethylene glycol (PEG) 4000, and crystals of mutant R240A were grown in a reservoir solution containing 0.1 M Hepes sodium, pH 7.5, 10% (*v/v*) 2-Propanol, 20% PEG 4000. Other mutants were crystallized under the same conditions as wild-type.

The crystals were cryoprotected by adding 20% (*v/v*) glycerol to each crystallization solution. The X-ray diffraction data of FdlA-NTD and various mutants were collected at beamlines BL17U1, BL18U1, BL19U1, and BL02U1 of the Shanghai Synchrotron Radiation Facility (SSRF) in China [42,43]. All diffraction data were processed using the program HKL2000 [44]. Data collection statistics are shown in Table S1.

The initial phase of FdlA-NTD was solved by single-wavelength anomalous diffraction (SAD) method using Autosol in the Phenix program (version 1.15.2) [45]. The structural model was automatically built through AutoBuild in the Phenix program. Structures of FdlA-NTD mutants were determined by molecular replacement (MR) method through Phaser-MR in the Phenix program using the WT FdlA-NTD structure as the initial model. All structures were refined through Refine in Phenix program and Coot (version 8.6.1) [46] alternately. The statistics of structural refinement were summarized in Table S1.

### 4.4. Circular Dichroism 

CD spectra were recorded by Chirascan Plus (Applied Photophysics Ltd., London, UK) and used to evaluate the structural stability of FdlA-NTD by detecting its secondary structural change under different conditions. Protein was diluted to a final concentration of 0.2 mg/mL. The pH stability was estimated by measuring the CD spectra of protein incubated in Britton-Robinson (B & R) buffer systems at different pH (5.0–12.0) for 17 h at room temperature. For thermal stability, FdlA-NTD was incubated at different temperatures for 120 min in a buffer containing 50 mM sodium phosphate buffer (pH 7.5), and the CD spectra were recorded starting at 5 min.

### 4.5. Enzymatic Activity Assay 

Fucoidan from *Kjellmaniella crassifolia* was used as the substrate of FdlA-NTD enzyme assay. *Kjellmaniella crassifolia* was cultured along the coast of Dalian, China and Kj-fucoidan was extracted following the extraction procedure reported in earlier literature [19,21]. The standard reaction mixture (200 μL) of enzymatic assay consists of 1% (*w*/*v*) Kj-fucoidan and appropriately diluted enzyme solution. The lyase activity of FdlA-NTD toward Kj-fucoidan was measured by monitoring the increase of 232 nm absorbance, which was caused by the production of 4, 5-unsaturated glucuronic acid-containing oligosaccharides, in the mixture. The extinction coefficient of the 4, 5-unsaturated bond at 232 nm was assumed as 5.5 L/(mmol·cm). One unit of the enzyme was defined as the amount of enzyme needed to catalyze the production of 1 μmol unsaturated oligosaccharides per minute, and the activity of one-milligram enzyme was defined as the specific activity of FdlA-NTD. Absorption at 232 nm was measured continuously at room temperature for 10min using a U-3900 UV-Visible spectrophotometer (Hitachi High-tech, Tokyo, Japan). Each measurement was repeated at least two times.

### 4.6. Biochemical Characterization of FdlA-NTD

To determine the optimal catalytic pH of FdlA-NTD, B & R buffer systems (pH 5.0–12.0) were used at a concentration of 50 mM for the reaction using 1% (*w*/*v*) Kj-fucoidan as the substrate. To determine the optimal temperature, reactions were performed at different temperatures, ranging from 10 °C to 70 °C. All enzymatic reactions under different temperatures were incubated for 1min, and then the reaction mixtures were boiled to stop the catalysis. To determine the thermal inactivation of FdlA-NTD, the reaction system was pre-incubated at 20 °C, 30 °C, 35 °C, and 40 °C, respectively, for varied time intervals (5 min to 120 min) at pH 7.5, and then chilled on ice for at least 10 min. The activities were measured under standard conditions (pH 7.5, 40 °C for 1 min). To determine the optimal NaCl concentration, the activity of FdlA-NTD was measured under standard conditions with different NaCl concentrations (0.092 M–2 M).

The effects of metal ions or chemical reagents (1 mM) on the catalytic activity of FdlA-NTD were determined by adding 1 mM of various metal ions or chemical reagents [Pb(CH_3_COO)_2_, NiSO_4_, MnSO_4_, CuSO_4_, BaCl_2_, CoCl_2_, CaCl_2_, MgCl_2_, FeSO_4_, Fe_2_(SO_4_)_3_, KCl, LiCl, SDS, EDTA, β-mercaptoethanol, iodoacetamide] to the standard enzyme assay system as above. Since phosphate in B & R buffer might impact the assay, 50 mM Tris-HCl (pH 7.5) was used instead. The system without supplying metal ions or chemical reagents was used as the control.

Kinetic parameters were determined under initial rate conditions using non-linear regression analysis of the Michaelis–Menten equation. The lyase activity was measured at room temperature using Kj-fucoidan as substrate at concentrations ranging from 0.2 to 2% (*w*/*v*) in a 50 mM B & R buffer (pH 7.5) after being incubated for 60 s. 

### 4.7. Analysis of Degradation Products

The molecular weight (MW) of Kj-fucoidan is within a range since it is a mixture of heterogeneous polysaccharides. To estimate the average MW of Kj-fucoidan, the samples were analyzed by High-Performance Gel Permeation Chromatography (HPGPC) with TSK GEL GMPWXL column, and the polysaccharides were eluted with mobile phase containing double distilled water at a flow rate of 0.5 mL/min and detected by Evaporative Light-scattering Detector (Acchrom, Beijing, China). The average MW of Kj-fucoidan was estimated as 80 kDa.

High-performance liquid chromatography (HPLC) analysis was used to purify the degradation products of Kj-fucoidan cleaved by FdlA-NTD. FdlA-NTD was incubated with 1% (*w*/*v*) Kj-fucoidan in 50 mM sodium phosphate buffer (pH 7.5) for 3 h at 37 °C, and the enzyme was then inactivated by heating in a water bath at 100 °C for 10 min. The reaction mixture was centrifuged at 12,000 rpm for 10 min, and the supernatant was subjected to high-performance liquid chromatography analysis. HPLC analysis was performed on an Acchrom S6000 HPLC system (Acchrom Technologie, Dalian, China) and the separation was performed on an Acchrom XAmide column (4.6 mm × 250 mm, 5 μm, Acchrom Technologies, Dalian, China). The mobile phase consisted of water (A), acetonitrile (B), and ammonium formate (C) with the following elution gradients: mobile phase A from 0 to 40%, mobile phase B from 90% to 50%, and mobile phase C at 10% within 40 min, at a flow rate of 1.5 mL/min and a column temperature of 40 °C. The enzymatic digestion products were detected at 232 nm with a UV detector. 

Mass spectrometric (MS) analysis was used to identify the degradation products of FdlA-NTD. The purified enzymatic digestion products were mixed with the matrix DHB and analyzed by a matrix-assisted laser resolved ionization-time of flight mass spectrometer (UltraflextremeTM MALDI-TOF/TOF, Brucker, Karlsruhe, Germany) in reflection mode.

### 4.8. Microscale Thermophoresis Assay

Microscale thermophoresis (MST) assay was performed on NT.115 Monolith instrument from NanoTemper Technologies using standard treated capillaries (NanoTemper, Munich, Germany). The purified wild-type and mutant proteins (10 μM) were labeled using a Protein Labeling Kit RED-NHS 2nd Generation. The substrate Kj-fucoidan (45 μM) was prepared in a ligand buffer containing 25 mM Hepes pH 7.5, 100 mM NaCl, and done two-fold dilution in series. For the MST assay, 150 nM labeled protein was incubated with a series of substrates in a ligand buffer containing 0.05% Tween-20 for 5 min in NT.115 capillaries separately. Each capillary containing the mixed enzyme and substrate was tested by Monolish NT.115 at 25 °C, 20% excitation power, and medium MST power. Thermophoresis data were analyzed by MO. Affinity Analysis software (version 2.3, NanoTemper) [47]. Each measurement was repeated at least two times.

### 4.9. Molecular Docking 

Molecular docking analysis of FdlA-NTD with various oligosaccharides (substrates) was performed to identify the amino acid residues potentially critical for the active site formation and catalysis of FdlA-NTD. The structural coordinates of oligosaccharide molecules containing a sulfate group were built with a CHARMM-GUI server online (http://www.charmm-gui.org/, accessed on 15 July 2021) [48] and converted into mol2 format by Open Bable tool for Ledock program (version 1.0) [37,49]. A root-mean-squares deviation (RMSD) value and the number of binding poses were set to 1 and 500, respectively. All docked results were sorted by score energy ranking. The docking results ranking on the top of the list were chosen for analysis in the next step.

## 5. Conclusions

FdlA from *Flavobacterium* sp. SA-0082 is the first fucoidan lyase reported so far. Here, we determined the atomic-resolution crystal structures of the FdlA N-terminal catalytic domain. In addition, we performed extensive biochemical and enzymatic analysis on the wild-type and mutant forms of FdlA-NTD, revealing key residues essential for the catalysis. Compared with other β-helix PLs, FdlA-NTD possesses a similar overall folding, but considerably different active site and key residues that potentially serve as the catalytic acid and base in β-elimination reaction. Moreover, we revealed that FdlA-NTD uses a unique ‘groove-pocket’ for substrate binding and the alkaline pocket is suitable to accommodate a trisaccharide unit, thus rationalizing the observation that the final product of FdlA-NTD is exclusively trisaccharide. Together, our work identified the unique structural and catalytic features of FdlA-NTD, providing novel insights into the mode of action of PLs, and enriching our knowledge on the fucoidan-degrading enzymes. Our results may further aid the applied research in designing mutated forms of FdlA-NTD that would be used in producing specific products for industrial use.

## Data Availability

Atomic coordinates and crystallographic structure factors have been deposited in the protein data bank under accession codes: 7XZF (WT), 7XZ7 (D137A), 7XZ8 (K141), 7XZE (H176A), 7XZD (F179A), 7XZ9 (E236A), 7XZC (R240A), 7XZB (Y242A), 7XZA (Y242F). All other data supporting the finding in this study are available from the corresponding authors upon reasonable request.

## Supplementary Materials

The following supporting information can be downloaded at: https://www.mdpi.com/article/10.3390/md20080533/s1, Figure S1: Sequence alignment of FdlA and FdlB; Figure S2: Purification and characterization of FdlA-NTD; Figure S3: Structural superposition of two molecules in an asymmetric unit of FdlA-NTD crystal structure; Figure S4: Comparison of FdlA-NTD with representative members of other β-helix PL families; Figure S5: The sulfate groups and docked trisaccharide in the ‘groove-pocket’ region of FdlA-NTD; Figure S6: The MST curves of inactive mutants of FdlA-NTD with the substrate (Kj-fucoidan); Figure S7: Electron density maps of the mutated site in inactive mutants; Table S1: Diffraction data and refinement statistics of WT and mutants of FdlA-NTD.

## References

[1] Li B., Lu F., Wei X., Zhao R. (2008). Fucoidan: Structure and Bioactivity. Molecules.

[2] Yu L., Ge L., Xue C., Chang Y., Zhang C., Xu X., Wang Y. (2014). Structural study of fucoidan from sea cucumber *Acaudina molpadioides*: A fucoidan containing novel tetrafucose repeating unit. Food Chem..

[3] Anastyuk S.D., Shevchenko N.M., Nazarenko E.L., Dmitrenok P.S., Zvyagintseva T.N. (2009). Structural analysis of a fucoidan from the brown alga *Fucus evanescens* by MALDI-TOF and tandem ESI mass spectrometry. Carbohydr. Res..

[4] Ale M.T., Mikkelsen J.D., Meyer A.S. (2011). Important Determinants for Fucoidan Bioactivity: A Critical Review of Structure-Function Relations and Extraction Methods for Fucose-Containing Sulfated Polysaccharides from Brown Seaweeds. Mar. Drugs.

[5] Cao H., Mikkelsen M., Lezyk M., Bui L., Tran V., Silchenko A., Kusaykin M., Pham T., Truong B., Holck J. (2018). Novel Enzyme Actions for Sulphated Galactofucan Depolymerisation and a New Engineering Strategy for Molecular Stabilisation of Fucoidan Degrading Enzymes. Mar. Drugs.

[6] Zayed A., El-Aasr M., Ibrahim A.-R.S., Ulber R. (2020). Fucoidan Characterization: Determination of Purity and Physicochemical and Chemical Properties. Mar. Drugs.

[7] Usov A.I., Bilan M.I. (2009). Fucoidans—Sulfated polysaccharides of brown algae. Russ. Chem. Rev..

[8] Kwon P.S., Oh H., Kwon S.J., Jin W., Zhang F., Fraser K., Hong J.J., Linhardt R.J., Dordick J.S. (2020). Sulfated polysaccharides effectively inhibit SARS-CoV-2 in vitro. Cell Discov..

[9] Luthuli S., Wu S., Cheng Y., Zheng X., Wu M., Tong H. (2019). Therapeutic Effects of Fucoidan: A Review on Recent Studies. Mar. Drugs.

[10] Wijesekara I., Pangestuti R., Kim S.-K. (2011). Biological activities and potential health benefits of sulfated polysaccharides derived from marine algae. Carbohydr. Polym..

[11] Kusaykin M., Bakunina I., Sova V., Ermakova S., Kuznetsova T., Besednova N., Zaporozhets T., Zvyagintseva T. (2008). Structure, biological activity, and enzymatic transformation of fucoidans from the brown seaweeds. Biotechnol. J..

[12] Kusaykin M.I., Silchenko A.S., Zakharenko A.M., Zvyagintseva T.N. (2016). Fucoidanases. Glycobiology.

[13] Furukawa S.-I., Fujikawa T., Koga D., Ide A. (2014). Purification and Some Properties of Exo-type Fucoidanases fromVibriosp. N-5. Biosci. Biotechnol. Biochem..

[14] Lahrsen E., Liewert I., Alban S. (2018). Gradual degradation of fucoidan from *Fucus vesiculosus* and its effect on structure, antioxidant and antiproliferative activities. Carbohydr. Polym..

[15] Silchenko A.S., Rasin A.B., Zueva A.O., Kusaykin M.I., Zvyagintseva T.N., Rubtsov N.K., Ermakova S.P. (2021). Discovery of a fucoidan endo-4O-sulfatase: Regioselective 4O-desulfation of fucoidans and its effect on anticancer activity in vitro. Carbohydr. Polym..

[16] Vuillemin M., Silchenko A.S., Cao H.T.T., Kokoulin M.S., Trang V.T.D., Holck J., Ermakova S.P., Meyer A.S., Mikkelsen M.D. (2020). Functional Characterization of a New GH107 Endo-α-(1, 4)-Fucoidanase from the Marine Bacterium Formosa haliotis. Mar. Drugs.

[17] Zueva A.O., Silchenko A.S., Rasin A.B., Kusaykin M.I., Usoltseva R.V., Kalinovsky A.I., Kurilenko V.V., Zvyagintseva T.N., Thinh P.D., Ermakova S.P. (2020). Expression and biochemical characterization of two recombinant fucoidanases from the marine bacterium *Wenyingzhuangia fucanilytica* CZ1127(T). Int. J. Biol. Macromol..

[18] Sakai T., Kimura H., Kato I. (2002). A marine strain of flavobacteriaceae utilizes brown seaweed fucoidan. Mar. Biotechnol..

[19] Sakai T., Kimura H., Kato I. (2003). Purification of Sulfated Fucoglucuronomannan Lyase from Bacterial Strain of *Fucobacter marina* and Study of Appropriate Conditions for Its Enzyme Digestion. Mar. Biotechnol..

[20] Takayama M., Koyama N., Sakai T., Kato I. (2002). Enzymes Capable of Degrading a Sulfated-Fucose-Containing Polysaccharide and Their Encoding Genes. U.S. Patent.

[21] Sakai T., Kimura H., Kojima K., Shimanaka K., Ikai K., Kato I. (2003). Marine Bacterial Sulfated Fucoglucuronomannan (SFGM) Lyase Digests Brown Algal SFGM into Trisaccharides. Mar. Biotechnol..

[22] Lombard V., Bernard T., Rancurel C., Brumer H., Coutinho Pedro M., Henrissat B. (2010). A hierarchical classification of polysaccharide lyases for glycogenomics. Biochem. J..

[23] Xu F., Wang P., Zhang Y.-Z., Chen X.-L., Zhou N.-Y. (2018). Diversity of Three-Dimensional Structures and Catalytic Mechanisms of Alginate Lyases. Appl. Environ. Microbiol..

[24] Garron M.-L., Cygler M. (2014). Uronic polysaccharide degrading enzymes. Curr. Opin. Struct. Biol..

[25] Garron M.L., Cygler M. (2010). Structural and mechanistic classification of uronic acid-containing polysaccharide lyases. Glycobiology.

[26] Baumgartner S., Hofmann K., Chiquet-Ehrismann R., Bucher P. (1998). The discoidin domain family revisited: New members from prokaryotes and a homology-based fold prediction. Protein. Sci..

[27] Veith P.D., Nor Muhammad N.A., Dashper S.G., Likić V.A., Gorasia D.G., Chen D., Byrne S.J., Catmull D.V., Reynolds E.C. (2013). Protein Substrates of a Novel Secretion System Are Numerous in the Bacteroidetes Phylum and Have in Common a Cleavable C-Terminal Secretion Signal, Extensive Post-Translational Modification, and Cell-Surface Attachment. J. Proteome Res..

[28] Hayakawa K., Guo L., Terentyeva E.A., Li X.-K., Kimura H., Hirano M., Yoshikawa K., Nagamine T., Katsumata N., Ogata T. (2006). Determination of specific activities and kinetic constants of biotinidase and lipoamidase in LEW rat and *Lactobacillus casei* (*Shirota*). J. Chromatogr. B.

[29] Yoder M.D., Lietzke S.E., Jurnak F. (1993). Unusual structural features in the parallel beta-helix in pectate lyases. Structure.

[30] Scavetta R.D., Herron S.R., Hotchkiss A.T., Kita N., Keen N.T., Benen J.A., Kester H.C., Visser J., Jurnak F. (1999). Structure of a plant cell wall fragment complexed to pectate lyase C. Plant Cell.

[31] Huang W., Matte A., Li Y., Kim Y.S., Linhardt R.J., Su H., Cygler M. (1999). Crystal structure of chondroitinase B from Flavobacterium heparinum and its complex with a disaccharide product at 1.7 A resolution. J. Mol. Biol..

[32] Petersen T.N., Kauppinen S., Larsen S. (1997). The crystal structure of rhamnogalacturonase A from Aspergillus aculeatus: A right-handed parallel beta helix. Structure.

[33] Alahuhta M., Taylor L.E., Brunecky R., Sammond D.W., Michener W., Adams M.W., Himmel M.E., Bomble Y.J., Lunin V. (2015). The catalytic mechanism and unique low pH optimum of *Caldicellulosiruptor bescii* family 3 pectate lyase. Acta Crystallogr. D Biol. Crystallogr..

[34] Lyu Q., Zhang K., Shi Y., Li W., Diao X., Liu W. (2019). Structural insights into a novel Ca^2+^-independent PL-6 alginate lyase from Vibrio OU02 identify the possible subsites responsible for product distribution. Biochim. Biophys. Acta (BBA) Gen. Subj..

[35] Luis A.S., Briggs J., Zhang X., Farnell B., Ndeh D., Labourel A., Baslé A., Cartmell A., Terrapon N., Stott K. (2017). Dietary pectic glycans are degraded by coordinated enzyme pathways in human colonic Bacteroides. Nat. Microbiol..

[36] Itoh T., Nakagawa E., Yoda M., Nakaichi A., Hibi T., Kimoto H. (2019). Structural and biochemical characterisation of a novel alginate lyase from *Paenibacillus* sp. str. FPU-7. Sci. Rep..

[37] Wang Z., Sun H., Yao X., Li D., Xu L., Li Y., Tian S., Hou T. (2016). Comprehensive evaluation of ten docking programs on a diverse set of protein–ligand complexes: The prediction accuracy of sampling power and scoring power. Phys. Chem. Chem. Phys..

[38] Davies G.J., Wilson K.S., Henrissat B. (1997). Nomenclature for sugar-binding subsites in glycosyl hydrolases. Biochem. J..

[39] Xu F., Chen X.-L., Sun X.-H., Dong F., Li C.-Y., Li P.-Y., Ding H., Chen Y., Zhang Y.-Z., Wang P. (2020). Structural and molecular basis for the substrate positioning mechanism of a new PL7 subfamily alginate lyase from the arctic. J. Biol. Chem..

[40] Dong F., Xu F., Chen X.-L., Li P.-Y., Li C.-Y., Li F.-C., Chen Y., Wang P., Zhang Y.-Z. (2019). Alginate Lyase Aly36B is a New Bacterial Member of the Polysaccharide Lyase Family 36 and Catalyzes by a Novel Mechanism With Lysine as Both the Catalytic Base and Catalytic Acid. J. Mol. Biol..

[41] Kokoska R.J., McCulloch S.D., Kunkel T.A. (2003). The Efficiency and Specificity of Apurinic/Apyrimidinic Site Bypass by Human DNA Polymerase η and *Sulfolobus solfataricus* Dpo4. J. Biol. Chem..

[42] Wang Q.-S., Zhang K.-H., Cui Y., Wang Z.-J., Pan Q.-Y., Liu K., Sun B., Zhou H., Li M.-J., Xu Q. (2018). Upgrade of macromolecular crystallography beamline BL17U1 at SSRF. Nucl. Sci. Tech..

[43] Zhang W.-Z., Tang J.-C., Wang S.-S., Wang Z.-J., Qin W.-M., He J.-H. (2019). The protein complex crystallography beamline (BL19U1) at the Shanghai Synchrotron Radiation Facility. Nucl. Sci. Tech..

[44] Otwinowski Z., Minor W. (1997). Processing of X-ray diffraction data collected in oscillation mode. Methods Enzymol..

[45] Adams P.D., Grosse-Kunstleve R.W., Hung L.W., Ioerger T.R., McCoy A.J., Moriarty N.W., Read R.J., Sacchettini J.C., Sauter N.K., Terwilliger T.C. (2002). PHENIX: Building new software for automated crystallographic structure determination. Acta Crystallogr. D Biol. Crystallogr..

[46] Emsley P., Cowtan K. (2004). Coot: Model-building tools for molecular graphics. Acta Crystallogr. Sect. D Biol. Crystallogr..

[47] Scheuermann T.H., Padrick S.B., Gardner K.H., Brautigam C.A. (2016). On the acquisition and analysis of microscale thermophoresis data. Anal. Biochem..

[48] Park S.J., Lee J., Qi Y., Kern N.R., Lee H.S., Jo S., Joung I., Joo K., Lee J., Im W. (2019). CHARMM-GUI Glycan Modeler for modeling and simulation of carbohydrates and glycoconjugates. Glycobiology.

[49] O’Boyle N.M., Banck M., James C.A., Morley C., Vandermeersch T., Hutchison G.R. (2011). Open Babel: An open chemical toolbox. J. Cheminform..

