# Supplementary material for: Structural and Biochemical Analysis Reveals Catalytic Mechanism of Fucoidan Lyase from Flavobacterium sp. SA-0082
Source: Mar Drugs. 2022 Aug 20;20(8):533. doi: 10.3390/md20080533 (PMC9410043; doi:10.3390/md20080533)
Supplement: Supplementary file 1 [file marinedrugs-20-00533-s001.zip › marinedrugs-1859809-supplementary.pdf]

Figure S1. Sequence alignment of FdlA and FdlB.

The sequences of FdlA and FdlB (GenBank No. AAO00510.1 and AAO00511.1) from *Flavobacterium* sp. SA-0082 are aligned. The strictly conserved residues are shaded in red. The signal peptide region, the F5/8 type C domain and the por secretion tail region of FdlA were indicated by black, green and pink line, respectively. The por secretion tail region of FdlB was indicated by blue line.

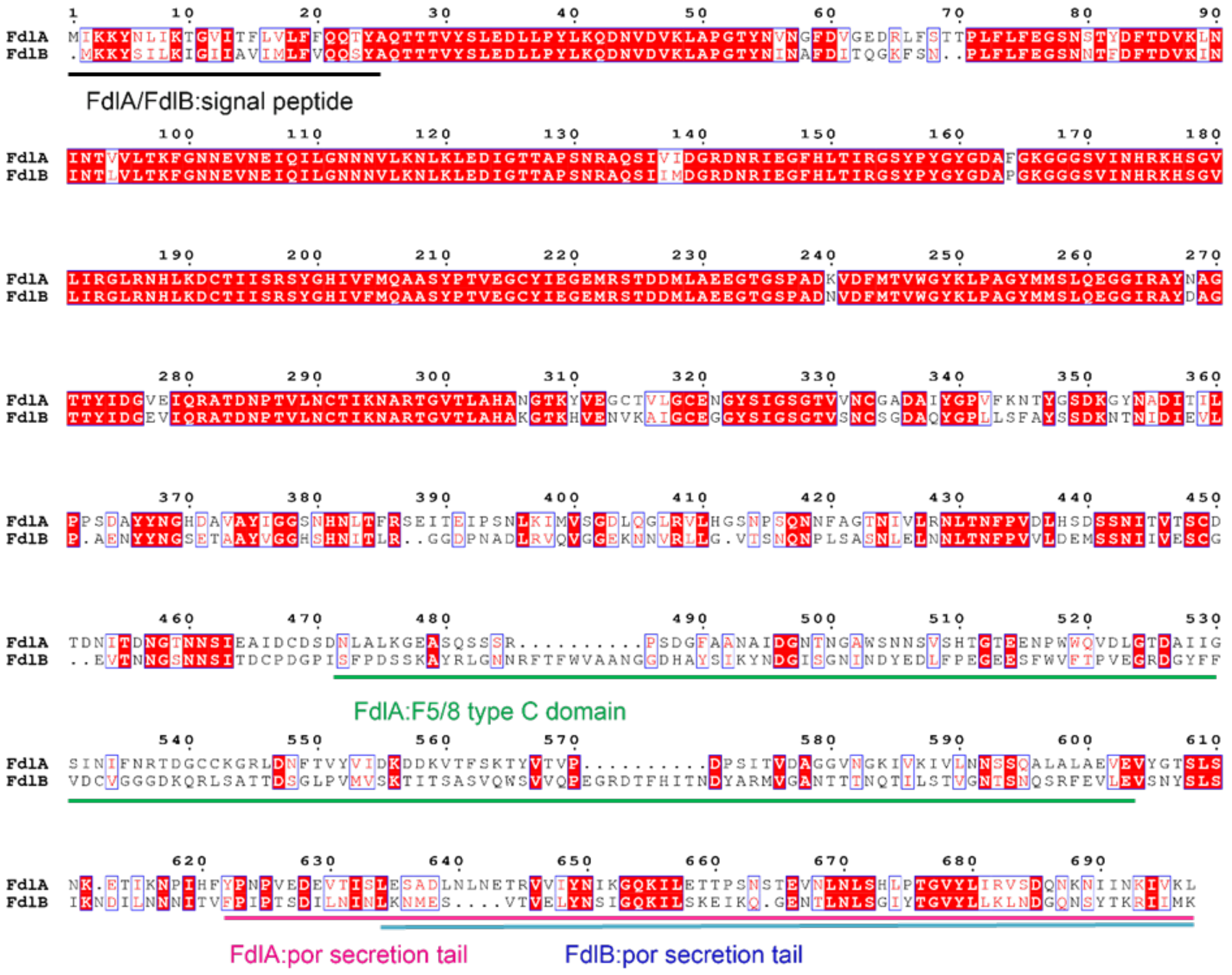

Figure S2. Purification and characterization of FdlA-NTD.

(A) Purification of FdlA-NTD by size exclusion chromatography.

(B) SDS-PAGE of recombinant FdlA-NTD (WT and mutants). The molecular weight of markers is labeled.

(C) CD spectra of FdlA-NTD after incubating at different temperatures within 120 min.

(D) CD spectra of FdlA-NTD after incubating at different pH for 17 h.

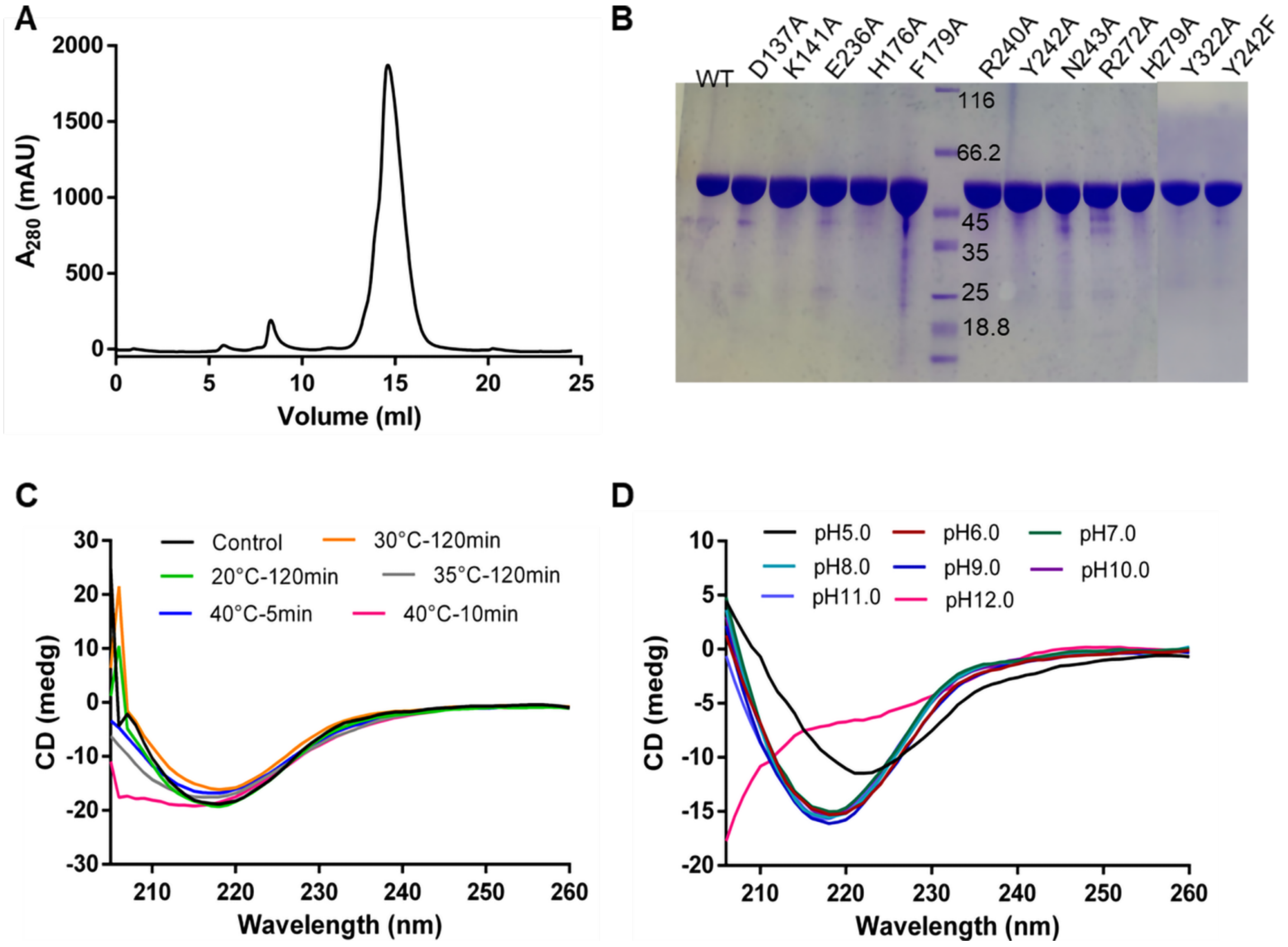

Figure S3. Structural superposition of two molecules in an asymmetric unit of FdlA-NTD crystal structure. Two molecules are coloured green and cyan, respectively.

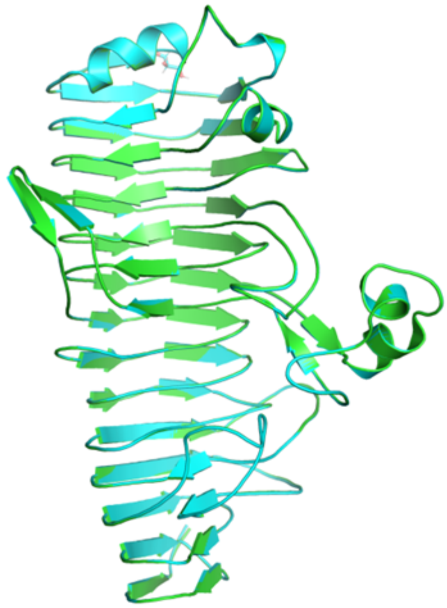

Figure S4. Comparison of FdlA-NTD with representative members of other β-helix PL families.

(A-E) Comparison of the sequence, overall structure and active site of FdlA-NTD with representative β-helix PL proteins from PL1 (pectate lyase, PDB code 2ewe) (A), PL3 (pectate lyase, PDB code 4z04 and 4ew9) (B), PL6 (alginate lyase, PDB code 6a40 and 6itg) (C), PL9 (pectate lyase, PDB code 5ols and 5olq) (D), and PL31 (alginate lyase, PDB code 6kfn) (E). The overall structures are shown in cartoon mode and colored green, cyan, yellow, pink, magenta and blue for FdlA-NTD, 2ewe, 4z04, 6a40, 5olr and 6kfn, respectively. The electrostatic surface of the active site of other PL proteins are shown in the zoom-in panel. The substrate and key residues are shown in stick mode, while metal ions are shown as spheres. The sequence alignment of FdlA-NTD with β-helix PLs are shown in lower panel. The catalytic residues of β-helix PLs are marked with circles and not conserved in FdlA-NTD.

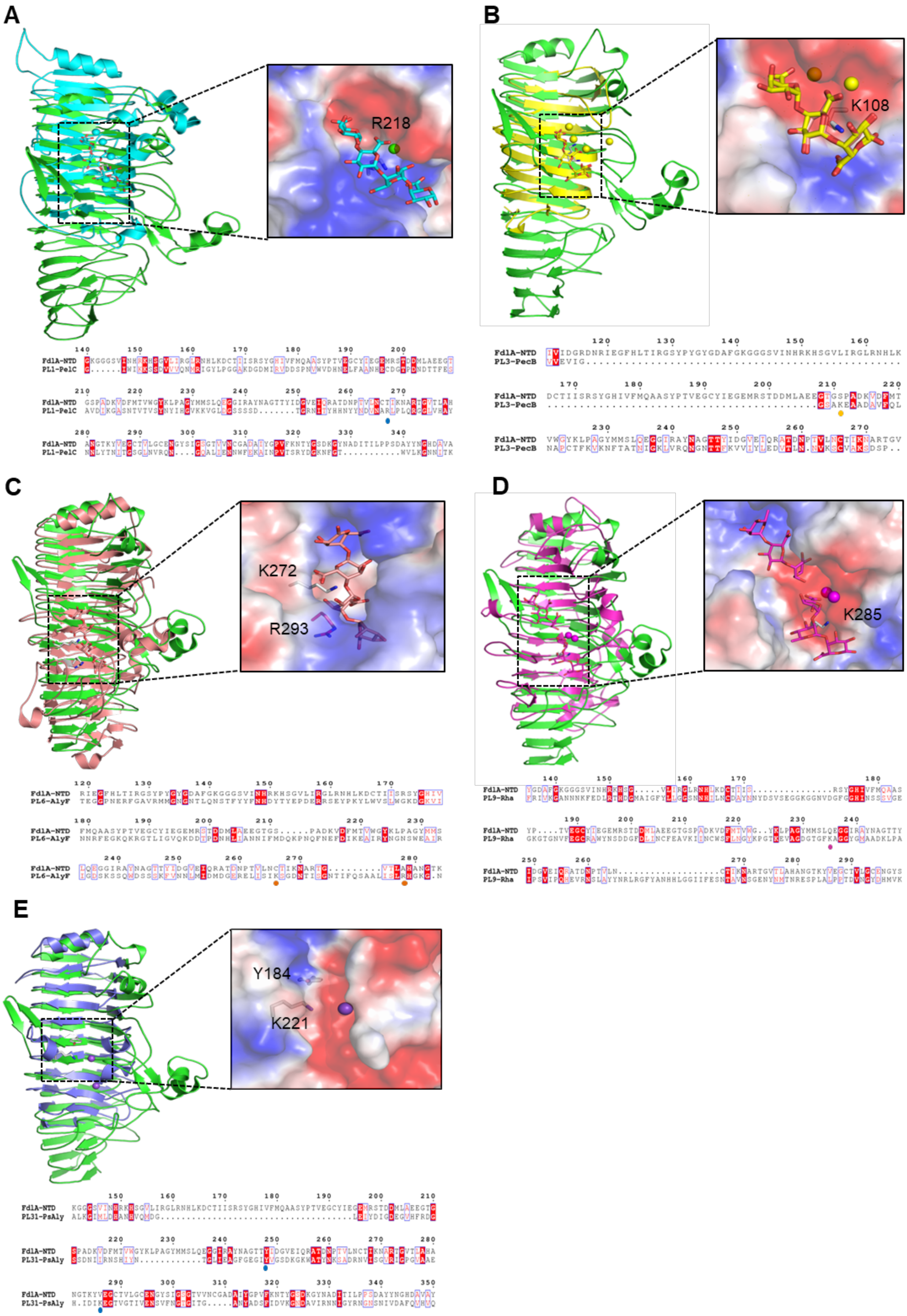
A
R218
B
K108
C
K272
R293
D
K285
E
Y184
K221

Figure S5. The sulfate groups and docked trisaccharide in the 'groove-pocket' region of FdlA-NTD.

(A) 2Fo-Fc (blue mesh) and Fo-Fc (green mesh) electron density maps of three sulfate groups contoured at 1.0 and 2.8 σ, respectively.

(B) The alkaline pocket is capable of accommodating a monosulfated trisaccharide molecule.

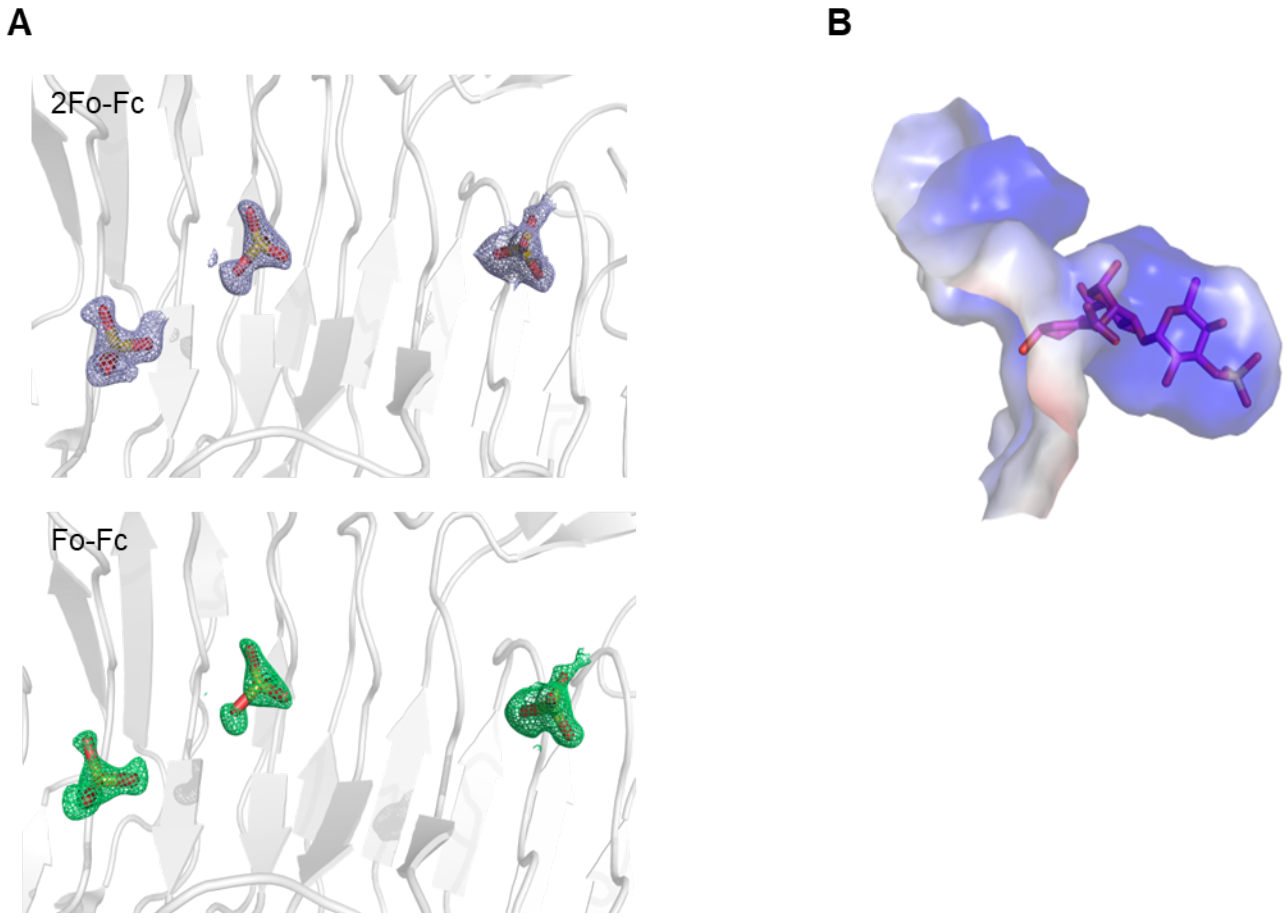

Figure S6. The MST curves of inactive mutants of FdlA-NTD with the substrate (Kj-fucoidan).

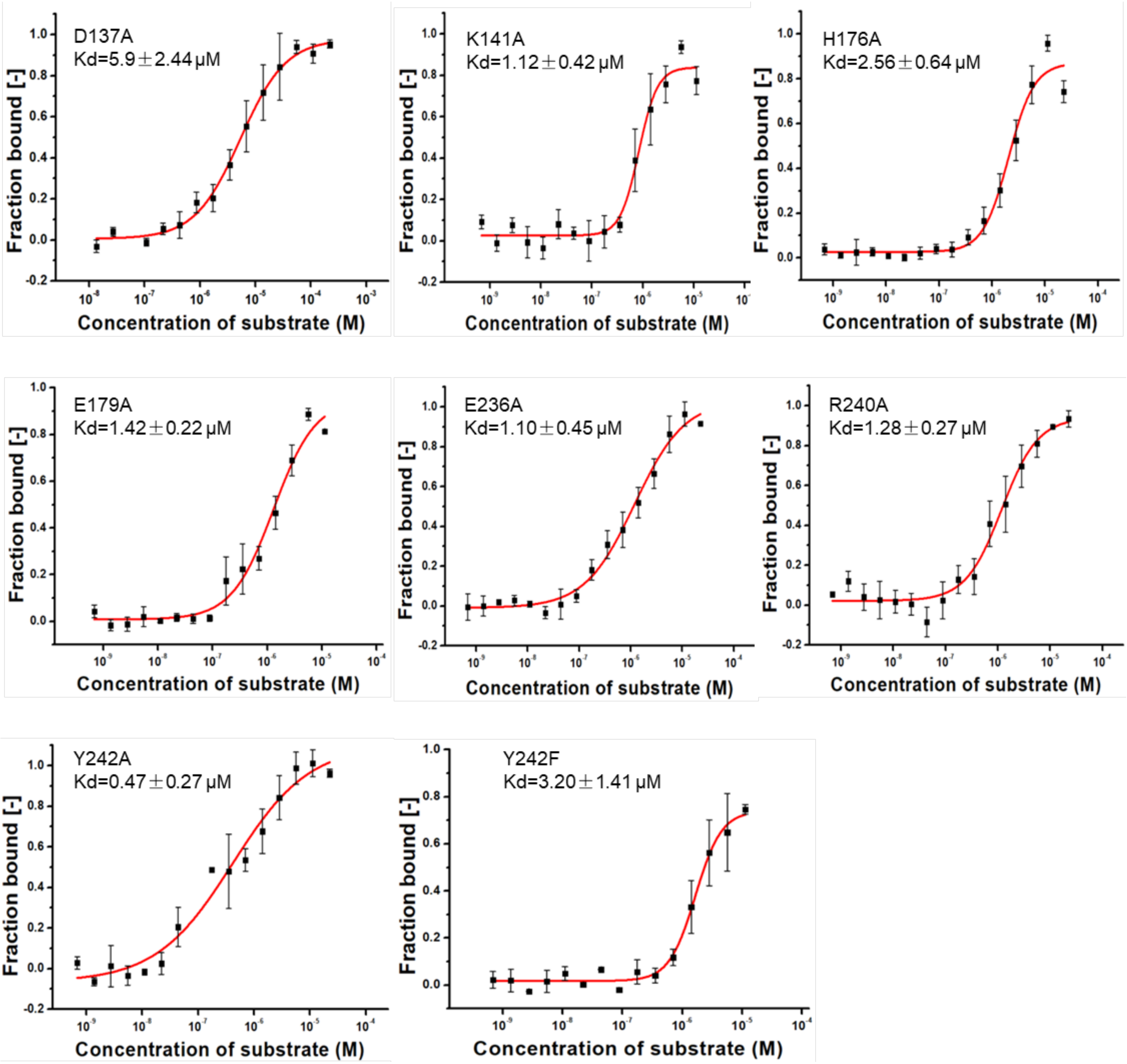

Figure S7. Electron density maps of the mutated site in inactive mutants.
(A-H) 2Fo-Fc (blue mesh, contoured at 1σ) and the negative Fo-Fc (red mesh, contoured at 3σ) electron density maps of Y242A (A), Y242F (B), K142A (C), R240A (D), F179A (E), H176A (F), E236A (G) and D137A (H) mutants, showing both the original residue in WT (green sticks) and the mutated residues in mutants (colored sticks).

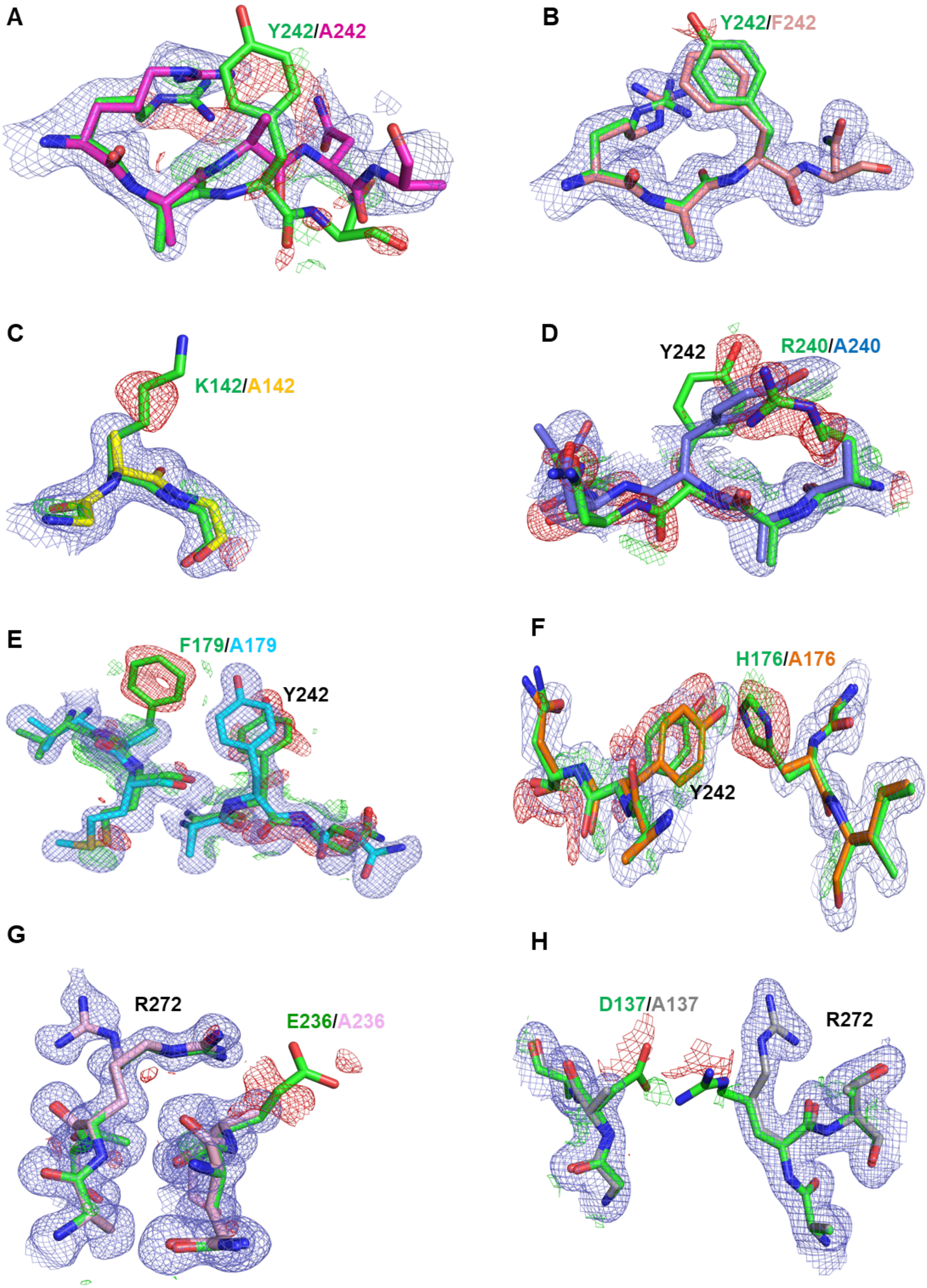

Table S1. Diffraction data and refinement statistics of WT and mutants of FdlA-NTD.

	WT	Se-SAD	D137A	K141A	H176A	F179A	E236A	R240A	Y242A	Y242F
**PDB code**	7XZF		7XZ7	7XZ8	7XZE	7XZD	7XZ9	7XZC	7XZB	7XZA
**Data collection**										
Wavelength (Å)	0.9785	0.9785	0.9792	0.9792	0.9792	0.9791	0.9791	0.9792	0.9792	0.9792
Space group	P1	P1	P1	$P6_1$	P1	P1	P1	P1	P1	P1
Cell dimensions										
a, *b*, *c* (Å)	54.0, 58.2, 88.3	52.9, 57.9, 88.3	54.5, 58.4, 88.3	90.4, 90.4, 123.6	54.0, 58.2, 88.5	90.2, 101.4, 92.2	53.8, 58.0, 87.9	53.8, 58.0, 88.1	53.6, 57.7, 88.2	54.1, 58.2, 88.2
α, β, γ (°)	90.3, 101.6, 92.0	90.3, 78.5, 87.9	90.2, 101.9, 92.2	90.0, 90.0, 120.0	90.2, 101.4, 92.2	90.0, 101.4, 92.0	90.4, 101.6, 92.0	90.2, 78.7, 87.9	90.1, 101.3, 92.0	90.2, 101.7, 92.1
Resolution (Å)	86-1.25 (1.27-1.25)[a]	50-1.80 (1.86-1.80)	58-1.98 (2.00-1.98)	34-1.89 (1.95-1.89)	50-1.80 (1.86-1.80)	58-1.65 (1.74-1.65)	50-1.54 (1.59-1.54)	50-1.70 (1.80-1.70)	27-2.25 (2.33-2.25)	86-2.08 (2.19-2.08)
R_{merge}[b]	0.062 (0.781)	0.201 (0.855)	0.079 (0.479)	0.156 (0.854)	0.070 (0.542)	0.084 (0.276)	0.171 (0.477)	0.090 (0.388)	0.163 (0.401)	0.120 (0.238)
$I/\sigma(I)$	8.9 (5.2)	10.3 (1.9)	6.7 (2.3)	9.7 (0.8)	24.3 (2.4)	6.0 (2.2)	5.3 (2.5)	7.2 (4.0)	3.3 (2.3)	3.9 (2.8)
Completeness (%)	95.1 (92.7)	96.5 (93.6)	91.0 (92.2)	99.1 (92.9)	92.5 (93.9)	71.1 (83.5)	96.3 (94.1)	93.8 (68.2)	95.5 (87.5)	91.0 (94.6)
Redundancy	2.0 (2.0)	10.5 (10.3)	3.8 (3.8)	19.9 (18.3)	6 (4.9)	2.3 (1.8)	3.4 (3.1)	3.7 (3.6)	3.3 (3.4)	1.7 (1.7)
**Refinement**										
Resolution (Å)	25-1.30		33-1.98	23-1.89	53-1.80	28-1.75	35-1.54	48-1.70	27-2.25	28-2.08
No. reflections	246,129		67,726	45,432	91,196	75,469	148,432	107,676	46,797	57,259
R_{work} / R_{free}	0.136/ 0.158		0.156/ 0.198	0.180/ 0.212	0.158/ 0.189	0.169/ 0.195	0.164/ 0.188	0.164/ 0.192	0.185/ 0.220	0.173/ 0.226
No. atoms										
Protein	7,188		6,706	3,415	6,819	6,820	6,879	6,833	6,737	6,850
Ligand	61				20	5			10	
Water	962		660	274	887	988	1208	725	622	582
B-factors ($Å^2$)										
Protein	11.0		31.8	41.8	17.6	15.3	10.6	19.4	17.9	17.6
Ligand	24.0				42.5	18.2			59.6	
Water	25.9		40.2	51.2	33.7	28.3	27.0	30.5	28.4	26.0
R.m.s. deviations										
bond length (Å)	0.014		0.018	0.007	0.014	0.009	0.006	0.012	0.005	0.008
bond angle (°)	0.4		1.45	0.92	1.12	1.07	0.89	1.09	0.89	0.95
Ramachandran Plot										
Favoured (%)	97.2		96.7	96.8	96.7	96.6	96.9	97.1	96.7	96.7
Allowed (%)	2.83		3.3	3.17	3.29	3.4	3.0	2.95	3.3	3.29
Outliers (%)	0.00		0.00	0.00	0.00	0.00	0.00	0.00	0.00	0.00

[a] Numbers in parentheses refer to data in the highest resolution shell.

[b] $R_{merge} = \sum_{hkl}\sum_i \mid I(hkl)_i \langle I(hkl)\rangle \mid / \sum_{hkl}\sum_i \langle I(hkl)_i\rangle$
